# Unlocking *cis*-regulatory landscapes across 500 million years of evolution and disease mechanisms

**DOI:** 10.1093/nargab/lqag107

**Published:** 2026-09-15

**Authors:** Tássia Mangetti Gonçalves, Casey L Stewart, Samantha D Baxley, Jason Xu, Kevin Boyer, Bijesh George, Daofeng Li, Chengran Yang, Harrison W Gabel, Xianhua Piao, Carlos Cruchaga, Yang E Li, Ting Wang, Oshri Avraham, Guoyan Zhao

**Affiliations:** Department of Genetics, Washington University School of Medicine, St. Louis, MO 63110, United States; Department of Cellular Biology, University of Georgia, Athens, GA 30602, United States; Department of Cellular Biology, University of Georgia, Athens, GA 30602, United States; Department of Computer Science, Missouri University of Science & Technology, Rolla, MO 65409, United States; Department of Genetics, Washington University School of Medicine, St. Louis, MO 63110, United States; Department of Genetics, Washington University School of Medicine, St. Louis, MO 63110, United States; Department of Genetics, Washington University School of Medicine, St. Louis, MO 63110, United States; Department of Psychiatry, Washington University School of Medicine, St. Louis, MO 63110, United States; Department of Neuroscience, Washington University School of Medicine, St. Louis, MO 63110, United States; Division of Neonatology, Department of Pediatrics, University of California, San Francisco, CA 94143,United States; Newborn Brain Research Institute, University of California, San Francisco, CA 94143,United States; Weill Institute for Neuroscience, University of California, San Francisco, CA 94143,United States; Department of Genetics, Washington University School of Medicine, St. Louis, MO 63110, United States; Department of Psychiatry, Washington University School of Medicine, St. Louis, MO 63110, United States; Department of Neurology, Washington University School of Medicine, St. Louis, MO 63110, United States; Department of Genetics, Washington University School of Medicine, St. Louis, MO 63110, United States; Department of Neurosurgery, Washington University School of Medicine, St. Louis, MO 63110, United States; Department of Genetics, Washington University School of Medicine, St. Louis, MO 63110, United States; The Edison Family Center for Genome Sciences and Systems Biology, Washington University School of Medicine, St. Louis, MO 63110, United States; Department of Cellular Biology, University of Georgia, Athens, GA 30602, United States; Department of Genetics, Washington University School of Medicine, St. Louis, MO 63110, United States; Department of Neurology, Washington University School of Medicine, St. Louis, MO 63110, United States; Department of Pathology and Immunology, Washington University School of Medicine, St. Louis, MO 63110, United States

## Abstract

Genomic DNA encodes regulatory information that determines where, when, and to what extent genes are expressed. Theoretically, we should be able to identify these transcriptional “instructions” by examining genomic DNA sequence alone, yet this has remained challenging. Here we present the Vertebrate Regulatory MOdule Detector (VRMOD), a method that accurately predicts gene regulatory sequences using only the query genomic sequences. We applied VRMOD to 309 Ensembl genomes, generating a compendium of high-resolution, genome-position-fixed *cis-*regulatory modules without parameter tuning. We performed extensive computational evaluation and experimental validation of VRMOD predictions. Notably, VRMOD predicted three sub-enhancers within the human hs52 enhancer at the FTO locus from the VISTA database, including one missed by existing methods. Using a chicken embryo system and 3D tissue imaging, we showed that each sub-enhancer exhibits restricted spatiotemporal activity within specific subsets of tissues where the full enhancer is active. We further demonstrated VRMOD’s utility for identifying evolutionarily non-conserved enhancers, annotating regulatory sequences in non-model organisms, and identifying candidate disease-causal variants. Collectively, VRMOD provides a universal coordinate reference system for regulatory sequences across 309 vertebrate genomes and enables genome-wide annotation of non-coding regulatory elements in any vertebrate species using genomic sequence alone.

## Graphical Abstract

**Graphical Abstract:**
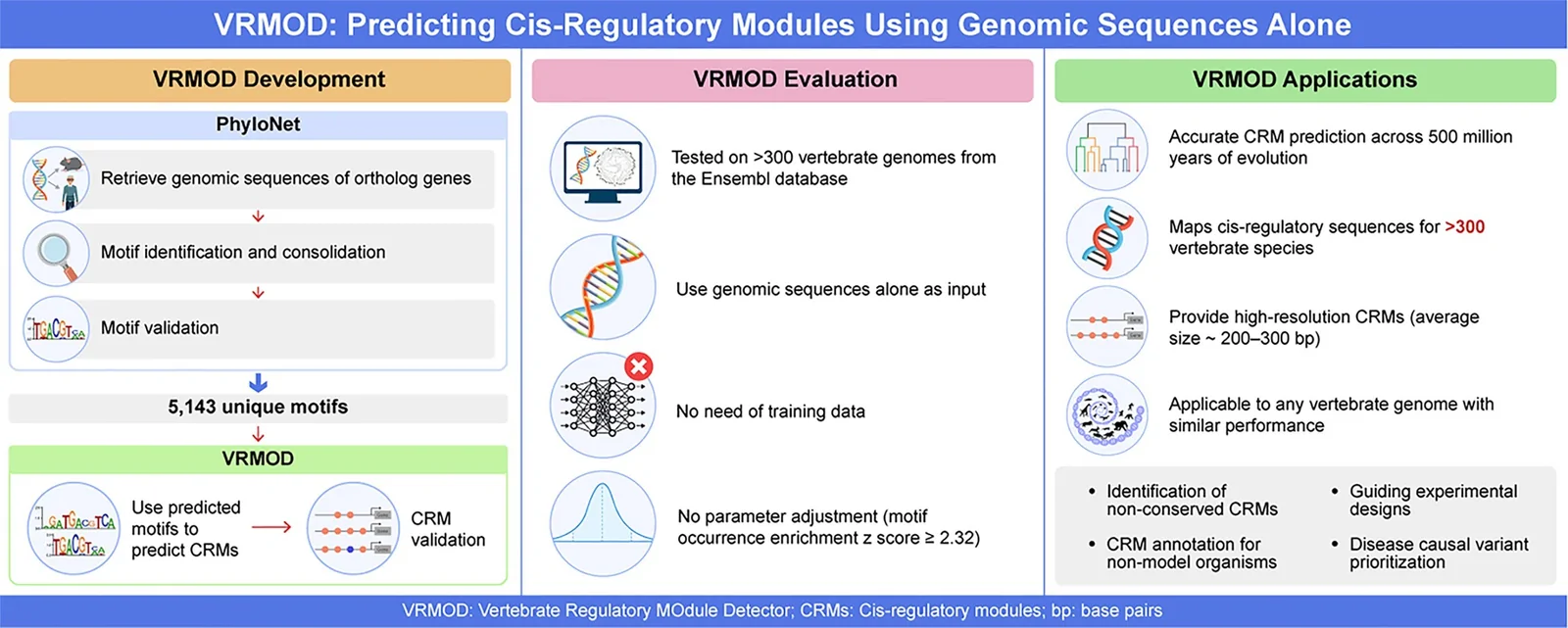

## Introduction

The first step of the central dogma of molecular biology describes the flow of genetic information from DNA to messenger RNA through transcription [1]. This process is regulated by transcription factors (TFs) that recognize short DNA sequence motifs known as transcription-factor-binding sites (TFBSs). Typically, multiple TFBSs are organized into modular clusters called *cis-*regulatory modules (CRMs), which ensure that each gene is transcribed at the correct time, in the appropriate cells, and at proper levels [2–4]. The DNA sequence of an organism encodes all the regulatory information necessary to direct tissue- and cell-type-specific gene expression during development, homeostasis, and responses to environmental and pathological stimuli [5]. In principle, it should therefore be possible to identify genomic regions that encode these transcriptional “instructions” by examining the DNA sequence alone. The concept of defining a *cis-*regulatory grammar—rules by which sequence determines transcriptional control—was proposed nearly three decades ago [6–8]. Since then, multiple models have been developed to describe the enhancer architecture, including the enhanceosome model [9], billboard model [10], TF-collective model [11], and dependency grammar [8]. Nevertheless, a universal *cis-*regulatory grammar capable of translating a DNA sequence into the spatial and temporal patterns of gene expression remains elusive [8, 12–15].

A sequence-alone-based regulatory grammar system capable of accurately pinpointing regulatory sequences in any genome will be of immense value to the field of genomics. Classically, CRMs have been defined through functional assays, primarily reporter gene assays, which demonstrate the ability of specific DNA fragments to modulate transcription. These experimentally defined CRMs (ExpCRMs) serve as the gold standard for functional CRMs. The VISTA Enhancer Browser remains the only database containing mammalian enhancers validated in transgenic mice [16, 17], comprising 673 and 998 experimentally confirmed enhancers from the mouse and human genomes, respectively. The activity of the enhancers has not been directly tested *in vivo* in mammals other than mice, underscoring the unique value of the VISTA collection. However, such assays are inherently low-throughput, labor-intensive, and costly [2, 18]. Recently, high-throughput empirical approaches, such as chromatin immunoprecipitation coupled to sequencing (ChIP-seq) [3, 19] for TFs and histone modification marks, DNase I hypersensitive site (DHS) sequencing (DNase-seq), assay for transposase-accessible chromatin using sequencing (ATAC-seq) [20, 21], STARR-seq [22], and massively parallel reporter assays (MPRAs) [23], have greatly facilitated candidate enhancer identification on a genome-wide scale [24]. Yet, without validation through gold-standard functional assays, the sequences identified by these methods remain “putative enhancers” [3, 25]. In fact, relatively high proportions of the biochemically or MPRA-supported candidate enhancers tested (up to ∼90% [26]) do not have a detectable influence on the expression of nearby genes [3, 25]. The reporter gene assay, often performed in cultured cells or transgenic animals, remains the definitive functional validation method (or gold-standard experimental approach) for enhancers [3, 25]. For these reasons, we will use the term “peaks” to refer to genomic regions identified by high-throughput empirical approaches, “enhancers” exclusively for DNA fragments shown to increase transcription in gold-standard experimental validation approaches, and “CRMs” for any DNA fragments with a demonstrated regulatory function in any category, including enhancers, silencers, insulators, locus control regions (LCRs), and post-transcriptional regulatory elements. Furthermore, emerging evidence also indicates that histone modification mark-based “putative enhancers” may miss some of the most active enhancers or other categories of enhancers that lack “canonical” H3K4me1 or H3K27ac marks [27–31]. Moreover, despite the vast amount of data generated by empirical methods, only a fraction of potential enhancers in the genome have been captured. It is currently not possible to sample every cell type, developmental stage, or environmental stimulus because of the limited availability of reagents, starting materials, and technologies [25, 32]. In fact, we will never be able to test all combinations of cell types, stages of development, and environmental factors that may influence gene expression. With the rapid influx of new genomes being assembled daily, accurate genome annotation, particularly of regulatory elements, has become a bottleneck [33], especially for non-model organisms lacking empirical data that can be used as training data for CRM prediction. Consequently, sequence-alone-based regulatory sequence inferences provide a promising avenue to bridge current knowledge gaps and to guide future experimental efforts in regulatory element testing and mechanistic investigation [34].

Over the past decades, many computational tools have been developed to predict functional CRMs, primarily in model organisms, such as nematodes, flies, mice, and humans [2, 32, 35]. These methods can be broadly classified into three main categories based on their input data requirements. First, comparative genomics approaches identify conserved non-coding DNA regions across species under the assumption that functionally important sequences are under more evolutionary constraints than sequences with less vital functions. However, not all CRMs are conserved, and this method fails to detect newly evolved regulatory modules [2, 32]. Second, motif-based methods (reviewed in Suryamohan *et al*. and Hardison *et al*. [2, 32]) require prior knowledge of constituent TF-binding motifs, limiting their applicability. Third, approaches that include more recent deep-learning methods [36–39] require substantial training data, such as known CRMs or genomic and epigenomic datasets [2, 32, 35]. Multiple machine-learning models have been developed to predict regulatory sequences from sequences alone [39–41]. These approaches leverage cross-species training and validation to enable tissue-specific regulatory prediction in additional species. One limitation of these methods is that they require regulatory genomics data from the tissue or cell type of interest in at least two species for training, which limits their applicability to tissues and cell types for which such matched training data are available. In addition, the performance of these models in non-mammalian vertebrates has not yet been evaluated. The true test of any enhancer grammar model is its ability to identify functional enhancers directly from DNA sequences alone [8, 42–44]. To the best of our knowledge, no existing algorithm has yet achieved accurate, cross-species prediction of regulatory sequences across all vertebrate genomes solely from genomic sequences, without relying on empirical training data.

Here, we present VRMOD (Vertebrate Regulatory MOdule Detector), a method that accurately predicts regulatory sequences directly from genomic sequences alone for any vertebrate species, without requiring parameter adjustment or empirical training data. VRMOD builds on an established algorithm originally developed for the *Caenorhabditis elegans* genome [45], applied here without modifications to the algorithm itself—only the input TFBS motifs were changed. Instead of worm-derived motifs, VRMOD uses a comprehensive set of TFBS motifs derived from human, rat, and mouse genomes. We applied VRMOD to 309 vertebrate genomes currently available in the Ensembl database [46], spanning an estimated phylogenetic distance of >500 million years [47, 48]. Because VRMOD requires only genomic sequences as inputs, the same algorithm and identical parameters were applied uniformly across all genomes, enabling consistent, high-resolution predictions even for species lacking empirical regulatory data. We performed extensive computational evaluation using available empirical datasets across all genomes used for TFBS detection (human, rat, and mouse) and additional organisms (chicken, bovine, pig, goat, zebrafish, and *C. elegans*). Importantly, because VRMOD does not use empirical data at any step of prediction, these independent datasets provide orthogonal validation of prediction accuracy. VRMOD accurately recovered a variety of currently known types of regulatory modules, providing a compendium of high-resolution CRMs (average size <235 bp) that are not limited to any tissue, cell type, developmental stage, or stimulus conditions. For humans and mice, we evaluated predicted CRMs using multiple sources of orthogonal datasets, including thousands of experimentally defined CRMs collected from the literature and the VISTA Enhancer Browser, 21 epigenomic data sets representing putative regulatory elements from Enhancer Atlas [49], ENCODE candidate *Cis*-Regulatory Elements (ENCODE2 and ENCODE4 cCREs) [50], the Cistrome Data Browser [51], and single-cell ATAC-seq data (scATAC-seq) from both the mouse brain atlas [52] and the human genome [53], encompassing hundreds of cell types, tissues, developmental contexts, and conditions. For chicken and bovine genomes, we used experimentally defined CRMs curated from the literature [54, 55] as gold-standard references. Additional genome-wide evaluations using zebrafish embryonic scATAC-seq peaks [56], datasets available from the Enhancer Atlas database, and the Functional Annotation of Animal Genomes (FAANG) Consortium [57] confirmed consistent performance across chicken, pig, rat, zebrafish, and *C. elegans*. Guided by VRMOD predictions, we experimentally dissected a large human enhancer from the VISTA database, identifying three refined sub-enhancers within the human enhancer hs52 at the FTO locus, which were missed by existing methods. Using a chicken embryo system combined with 3D tissue imaging, we demonstrated that each sub-enhancer exhibits restricted spatiotemporal activity within specific subsets of tissues where the full enhancer is active. Furthermore, VRMOD accurately predicted non-conserved CRMs that were missed by ENCODE cCREs. Finally, by integrating VRMOD prediction with expression quantitative trait loci (eQTLs) and experimental datasets, we identified candidate functional variants and regulatory elements likely to modulate disease susceptibility. Using this framework, we successfully identified candidate causal regulatory variants and a putative TF underlying Alzheimer’s disease (AD) pathogenesis. Collectively, our work provides a universal and stable common coordinate reference system for regulatory DNA across 309 vertebrate genomes, analogous to annotated gene models for protein-coding sequences. We have provided access to VRMOD software, the predicted CRMs across the 309 genomes, the experimental supporting evidence, and human CRMs with candidate neurodegenerative-disease-causal variants as part of the WashU Epigenome Browser, the UCSC Genome Browser public track hub, and open source repository. These collective resources provide a foundation for future medical, functional, and evolutionary research.

## Materials and methods

### Glossary

CRMfull refers to the complete set of CRMs predicted for a given genome.

CRM subset (CRMsub) refers to a length-restricted subset of CRMfull (length ≤250 bp) in a given genome whose total genomic coverage is sufficiently small to allow simulation of their distributions within the predicted non-functional sequence space. This enables generation of control DNA fragments (CTRL) that matched the CRMsub with identical number and length distributions, ensuring a fair and accurate comparison between functional CRMs (CRMsub) and predicted non-functional regions (CTRL).

CTRL refers to a set of genomic fragments that matched the CRMsub in both number and length distribution but were sampled from regions predicted by VRMOD to be non-functional.

ExpCRMs refer to experimentally validated CRMs identified through functional assays, primarily reporter gene assays, that demonstrate the ability of specific DNA fragments to modulate transcription. They serve as the gold standard for defining functional CRMs.

ExpSNPs refer to experimentally validated non-coding SNPs (ExpSNPs) for which changes led to changes in reporter gene expression levels.

### Genome sequence and orthologous genes

The unmasked genomic DNA sequences and annotation of mouse (*Mus musculus*) assembly GRCm38 (mm10) and human (*Homo sapiens*) assembly GRCh38 (hg38) from the Genome Reference Consortium were downloaded from the Ensembl website (https://nov2020.archive.ensembl.org/Mus_musculus/Info/Index and https://useast.ensembl.org/Homo_sapiens/Info/Index). The mouse genome was used as the anchor genome. Human and rat orthologs of mouse genes were downloaded from BioMart (https://useast.ensembl.org/info/data/biomart/index.html). Intergenic region sequences of up to 5 kb in length upstream of the start codon ATG of each gene in the corresponding genome were retrieved. In cases where the distance to the next upstream gene was <5 kb, only the available intergenic region was extracted. We chose to analyze this region because it is well-established that the majority of regulatory elements are located within this proximal upstream of genes [58–60]. This approach allows us to capture a significant portion of the regulatory landscape while maintaining a practical and computationally feasible scope for our analysis.

### PWM prediction using PhyloNet and motif consolidation

Using mouse genome as the anchor, we obtained a set of 18 215 genes with at least one ortholog in the rat or human genome. We retrieved up to 5 kb sequences upstream of the ATG codon for all of the genes. Each mouse gene and its orthologs formed a data entry in constructing a database, which includes all the orthologous gene sets. Each data entry was used as the input independently by PhyloNet [61] to query the database to identify conserved *cis-*regulatory elements that were associated with multiple genes. PhyloNet was run with options “-q 2000 -iq 500 -id 500 -s 4 -c1 -o2 -pf 50.” Up to 50 of the most significant position weight matrices (PWMs) from each query gene set were saved for further analysis. If an orthologous gene set contributed sequence motifs to the PWM, the mouse gene will be included in the gene list associated with the PWM. Because these initial predicted motifs in the PhyloNet output files are highly redundant, we took two steps to consolidate predicted motifs using the ALLR statistics [62] as described previously [45]. Briefly, the first step compares matrices in each query output file to consolidate matrices obtained from the same query sequences that significantly overlap. The unique PWMs obtained from each query gene at the first step were pooled together and further consolidated to generate the final set of 5143 distinct motifs (*P*-value < 10^−10^) with lengths between 5 and 30 bases (average 18 bp).

### Comparison with transcription factor PWMs in the TRANSFAC and CIS-BP databases

Predicted PWMs were compared with PWMs of CIS-BP (version 2.00) and TRANSFAC (version 10.2) [63, 64] using MatAlign-v4a (Wang and Stormo; http://stormo.wustl.edu/MatAlign/). Matrices that had an ALLR score >6.57 and the percentage of overlap between two matrices (OLAP score) >68.1% were considered redundant [45]. For each round of comparison, the best PWM was picked first (the one with the highest total ALLR score in the PhyloNet output). It was compared with the rest of the matrices using ALLR statistics, and any matrix that appeared redundant to the chosen matrix was removed. Then, the second-best one was picked, and the process was repeated until all the matrices had been analyzed.

### ChIP-Atlas database mouse transcription factor target enrichment analysis

ChIP-Atlas [65] has a collection of analysis results from 2540 ChIP-seq data sets, including TFs and their potential target genes, totaling 723 TFs from 369 different cell types/tissues for mouse (mm10) (ftp://ftp.biosciencedbc.jp/archive/chip-atlas/data/mm10/target). The peaks used in this study from ChIP-Atlas were ±5 kb from the transcription start site of RefSeq. The threshold of significance used to determine whether a target gene is enriched for a TF was >500 binding scores of MACS2. Significant overlap between predicted PWM-associated genes and TF targets from ChIP-Atlas was evaluated using a hypergeometric test. An FDR-corrected *P*-value < .05 was considered significant.

### Functional enrichment analysis of PWM-associated genes

Each PWM was associated with a set of genes that contributed at least one motif instance to the PWM during PhyloNet regulatory network construction. These PWM-associated gene sets were then analyzed using the ClusterProfiler R package (v4.0) [66] to identify Gene Ontology (GO) term enrichment and Kyoto Encyclopedia of Genes and Genomes (KEGG) pathway enrichment. Pathways with a false discovery rate (FDR)-adjusted *P*-value < .001 and a minimum of five genes were considered significantly enriched.

### Whole-genome-wide CRM identification

The 5143 mammalian PWMs identified by PhyloNet were used as input for the algorithm implemented in *C. elegans* Regulatory Module Detector (CERMOD) [45] to identify CRMs in the mouse and human genomes, respectively. First, Patser [67] was used to identify all predicted binding sites for the 5143 PWMs using default cutoff scores. Next, the algorithm calculated the average number of binding sites per position and the average number of binding sites per chromosome for each chromosome. The *z*-score for each position was calculated using the following formula:


\begin{eqnarray*}
{\mathrm{\mathit{ z}}} = \left( {{\mathrm{\mathit{ x}}} - \mu } \right)/\sigma .
\end{eqnarray*}

*x*: Represents the number of predicted binding events (over all 5143 PWMs) at a given chromosome position.

*μ*: Represents the average number of predicted binding events (over all 5143 PWMs) for the chromosome.

*σ*: Represents the standard deviation of the number of predicted binding events (over all 5143 PWMs) for the chromosome.

*z*: The resulting *z*-score, indicating how many standard deviations a position is away from the mean number of predicted binding events for a given chromosome.

Peak positions that have a *z*-score > 2.33 (corresponding to *P*-value < .01, one-tailed) were identified. For each peak position, we extended it in both 5′ and 3′ directions if the next *z*-score > 0 position is fewer than 30 bp away (the longest motif length). Peak positions used in the previous extension step were not extended. VRMOD identifies DNA regions that have TF binding sites significantly more than average (motif abundance *z*-score > 2.33, *P* < .01, one-tailed) and automatically determines the boundaries by *z*-score = 0 positions at each end of the DNA region. These regions correspond to putative regulatory CRMs.

### Method 1 for generation of control regions for VRMOD CRM prediction evaluation

We would like to obtain a set of control regions from each genome with the exact same genomic coverage and length distribution as the predicted CRMs to evaluate the performance of VRMOD. We first obtained regions not covered by CRMfull using BEDTools [68] complement function. Because it is impossible to predict the exact boundaries of CRMs, we filtered out regions <400 bp, which were fragments located between two adjacent predicted CRMs and could be part of the nearby functional CRMs. We also excluded regions with >50% “Ns.” These regions were predicted to be non-functional and were designated as non-functional regions. However, the predicted non-functional sequence space was insufficient to accommodate DNA fragments with the exact same genomic coverage and length distribution as the full set of CRMs (CRMfull). To address this, we defined a subset of CRMs (CRMsub) by excluding the longest predicted CRMs, resulting in a length-restricted subset of predicted CRM (CRMsub ≤ 250 bp). This adjustment provided sufficient non-functional sequence space to generate control DNA fragments that matched the CRMsub in both number and length distribution. The approach ensures a fair and accurate comparison between functional CRMs and non-functional regions. We created a custom in-house script “gene-slicing” (https://doi.org/10.5281/zenodo.21936089 or https://github.com/JasonXu314/gene-slicing) to trim the non-functional regions by 40 bp each end and to generate a DNA fragment distribution with exactly the same number and length as the CRMsub padding 5 bp between fragments. These regions were predicted to be non-functional, covering 21.8%, 20.8%, and 19.0% of the mouse, human, and zebrafish genomes, respectively, and had the exact same genomic coverage and similar distributions relative to annotated genes and transcripts as the CRMsub for all genomes. They were used as the control regions (referred to as CTRLs) to be compared with the CRMsub for odds ratio (OR) calculation to evaluate the accuracy of predicted CRMs. The R package ChIPseeker [69] was used to annotate CRMfull and control regions, as well as calculate feature genomic distribution.

### Alternative strategy (method 2) for generation of control regions for CRM prediction evaluation

Similar to the method 1 for generation of control regions, regions complementary to the CRMfull were obtained, and DNA fragments with >50% “Ns” were excluded. Regions <400 bp were also filtered to remove small regions that were located between two predicted CRMs. The remaining regions were referred to as the control regions. To obtain predicted CRMs with similar genomic coverage for a fair comparison, the longest CRMs were excluded with increasing length until a length cutoff was found that leads to subsets of predicted CRMs that have the same genomic coverage as the control regions (300 and 350 bp were used for mouse and human, respectively). In this method, the control regions had slightly higher genomic coverage than the subsets of predicted CRMs to give control regions a slight advantage of sensitivity.

### Comparison with experimentally defined CRMs, chromatin accessibility, and epigenomics data

Experimentally defined modules came from two sources. (i) We collected 97 experimentally defined CRMs for mouse and 60 modules for human genomes through an extensive literature search (Supplementary Tables S3 and S4). (ii) We downloaded 634 and 998 experimentally defined enhancers that exhibited enhancer activity [16] from VISTA Enhancer Browser for mouse and human, respectively. Human, mouse, rat, pig, chicken, zebrafish, and *C. elegans* predicted enhancers were obtained from Enhancer Atlas 2.0 (http://www.enhanceratlas.org/data/download/species_enh_csv.tar.gz). From the Cistrome database, chromatin accessibility (ATAC-seq and DNAse-seq) peaks and ChIP-seq histone marks (H3K27ac, H3K4me1, and H3K4me3) peaks were downloaded (http://cistrome.org/db/batchdata) for both mouse and human genomes. ATAC-seq Atlas is only available for mouse genome [70]. Additional ATAC-seq data for bovine, goat (Capra hircus, ARS1), chicken, and pig genomes were downloaded from the FAANG Consortium (https://data.faang.org/home) [104]. scATAC-seq peaks from zebrafish were downloaded from Zebrahub-Multiome (https://zebrahub.sf.czbiohub.org/epigenomics) [56]. ENCODE mouse and human cCREs [50] are available at the web-based server “SCREEN: Search Candidate *cis-*Regulatory Elements by ENCODE” (SCREEN; http://screen.encodeproject.org) and UCSC Genome Browser ENCODE cCREs Tracks. Mouse [52] and human [53] scATAC-seq can be accessed at their portal (http://catlas.org/mousebrain/ and http://catlas.org/humanenhancer/, respectively). Each dataset included peaks from hundreds of different tissues and cell types. Files from the same type of data were merged using *bedtools* merge to eliminate overlapping elements. Modules bigger than 2.5 kb after merger were eliminated before comparison. We define an experimentally defined module as being correctly predicted at two different cutoffs: (i) experimentally defined modules overlap with predicted CRMs by 50% of the length of the shorter one; (ii) they overlap by 1 bp. The OR, confidence interval, and *P*-value (chi-square independent test) were calculated comparing CRMsub and CTRL with each dataset of mouse, human, and zebrafish respectively. Peak-detection sensitivity and OR were calculated using the R package *fmsb*. The difference was considered significant with an OR > 1 and *P*-value < .05. The LiftOver tool was used to identify orthologous sequences between mouse and human genomes. CRMCs predicted by Ni *et al*. (Predicted CRMCs of *Homo sapiens*, version 2, hg38) were downloaded and intersected with VRMOD-predicted CRMs using bedtools intersect, and the fraction of features in each set overlapping by at least 1 bp with a feature in the other set was calculated.

### Evaluation of sequence conservation on CRM prediction sensitivity

To assess the effect of sequence conservation on prediction performance, we used human and mouse snATAC-seq peaks as independent, experimentally derived benchmark of regulatory activities. Conservation scores for each peak were calculated using the *GenomicScores* package [71]. Peaks were then evenly divided into 10 bins according to their conservation scores. For each bin, we assessed the overlap between snATAC-seq peaks and regions from CTRL, CRMfull, CRMsub, and ENCODE cCRE (≥50% overlap cutoff), followed by sensitivity calculation for each group.

### Evaluation of sequence GC content on CRM prediction sensitivity

We used human, mouse, and zebrafish snATAC-seq peaks as the evaluation sets. For each peak, we calculated GC content and divided the peaks evenly into 10 bins. Within each bin, we assessed overlap (≥50%) between snATAC-seq peaks and regions from CTRL, CRMfull, CRMsub, and ENCODE cCRE (for human and mouse genomes), then calculated the sensitivity for each group.

### Transcription factor binding site analysis

In order to find which TFBSs were disrupted by BIN1 ExpSNP rs4663105, we used the FASTA sequence of human CRM2743183 original sequence and disrupted sequence according to the BIN1 mutation (A > C). The two FASTA sequences were converted to a consensus sequence and used as input files together with seven PWMs from CIS-BP database that overlapped CRM2743183 and ExpSNP position to run Patser software. Motif sites of four PWMs were disrupted by the SNP: M09435, M00132, M09186, and M05877. These motifs are bound by the TFs TEAD4, OSR2, CDX1, and VDR, respectively.

### Animals and procedures

Fertilized White Leghorn chicken eggs (Hy-line North America, Mansfield, GA) were incubated at 38.5°C–39°C and 50%–80% humidity. A DNA solution of 2–5 mg/ml was injected into the lumen of the neural tube at HH stage 17–18 (E2.75–E3). Electroporation was performed using 3 × 50 ms pulses at 25 V, applied across the embryo using a 0.5-mm tungsten wire and a BTX electroporator (ECM 830). 100 units/ml penicillin in Hank’s Balanced Salt Solution was added on top of the embryos, and embryos were incubated for 24 h prior to analysis. All procedures involving chicken embryos were conducted in accordance with University of Georgia Institutional Animal Care and Use Committee (IACUC) guidelines. Chicken embryos were euthanized at embryonic day 4, prior to the stage at which avian embryos are considered capable of experiencing pain and are not regulated as live vertebrate animals until embryonic day 16. This work was therefore exempt from full IACUC protocol review.

### Embryo clearing

Whole embryos were excised and fixed in 4% Paraformaldehyde (PFA) overnight at 4°C with gentle shaking. Tissue was then washed using 1× phosphate buffered saline (PBS) 3 times for 2 h each at room temperature. Samples were placed in conical tubes, protected from light with foil, placed on an orbital shaker with gentle agitation, and soaked in following solutions: (i) Pre-delipidation: 50% CUBIC-L (T3740, TCI) diluted with double-distilled water at 37°C overnight, (ii) Delipidation: 100% CUBIC-L for 3 days at 37°C. (iii) Wash: rinsed in 1× PBS 3 times for 2 h each at room temperature. (iv) Post-fixation: fixed in 1% PFA at 4°C overnight, followed by 1 h in 1% PFA at 37°C. (v) Wash: rinsed in 1× PBS 3 times for 2 h each at room temperature. (iv) RI Match: incubated in 50% CUBIC-R (T3741, TCI) diluted with double-distilled water at room temperature overnight, followed by incubation in 100% CUBIC-R for 3 days at room temperature. Embryos were then stored in mounting solution (M3294, TCI) until imaging.

### Image acquisition and processing

Images of live embryos were taken under a fluorescent stereoscope (SMZ-745T Zoom Stereo Photo Microscope, Nikon), and images processed with Nikon NIS-Elements software. Cleared embryos were imaged with a light-sheet fluorescent microscope (Miltenyi BioTec), and image processing was done using Imaris v9.3 (BitPlane).

### DNA constructs

The hs52 element was amplified by PCR from a genomic human DNA (human foreskin fibroblasts) utilizing the primers [GCCAATTGCAATTTGGAATAACTTTCCCTACCC] and [GCGCTAGCTAAAAAGTGACCTGGGAAAACTCAG]. The hs52.1 element was amplified utilizing the primers [GCCAATTGCCCAGTAAATTGAGCATTACTCTAGG] and [GCGCTAGCAGGTGTTTGAGACCATGCCGCT]. The hs52.2 element was amplified utilizing the primers [GCCAATTGAACGCACCCTCTGTTCTTCAGT] and [GCGCTAGCGCACTAAGTACTATTATGTAGCACA]. The hs52.3 element was amplified utilizing the primers [GCCAATTGCAGGCTTGGAAATGGGGCCAGG] and [GCGCTAGCACGTGGCAGTGAAAACGAGTGG]. The enhancers were cloned into 5′MfeI/3′NheI sites of the Cre plasmid. The RPBSA: green fluorescent protein (GFP) plasmid was used as a positive control for electroporation (Addgene #60511).

### hs52

CAGGATAATGTGTGCACGGACAATTTATCCACTGTGAGATTGAAATGCTCAATTTGGAATAACTTTCCCTACCCAGTAAATTGAGCATTACTCTAGGATTCTGAGACAGAGAGAAAGCACAATTTTAAAAGCTTTGCAGAGTTCCTTTGTAATTAGTCGCAGCTTTCCTTGAATATTAATTTTCCCTGCATCCCTTTCAAGTGGTTGAGAGACTGTCTCTACAACTACAGAGATGCACCCTCAGAACAACGACAGCAAGCGGCATGGTCTCAAACACCTATGATTAAGTAGTCTACAGAACGCACCCTCTGTTCTTCAGTGCAGTGTGTAGCTTATCAGTGCAAACAGTTTAATATTTATGCTAAGAGGATTGTCAAAAGCAGCTTCTGTTGCTTTAATTCTTGTTTTAAATAAATAATGAGAACATTTAAACACATTACTCTTCTTGGGGCCCCGGGGTCAGCTAATCTTATTATTTATGAAGTGATGTGCTACAAATAGTACTTAGTGCATGTTAACAGACGCTATTATCAGGGCCGGATGCAGAGAGCTGAAGATATATTAGAATGTTATGTGTAATGTACGACGGATTGAGTGCATAGGATGCCGGTGTAGCAATTAACCACACTCGAAAATAGGTGTAAAGTTGAAGTATGTTTTCCCCGGGGGGATCCCCTCACCATTAATAATTCCCCAGAGAAGAAGATGTCTTTCAGTTAGGAACCCTCTCTACCATCAGGCTTGGAAATGGGGCCAGGATATTCCATTCTTTGATCTCTTCATAGTCAGTCCTACACAGTCAGAAGACAAATAGTGAGCATGACCACTTTTTAATTGATTTAGACAAAAATGGAGAGAAGGCGGGGGTGGAGGGAGGCACATGTGCAATGCTCCCAAGTGTCCTCATAGTGTTTGGTTTTGATCCACTCGTTTTCACTGCCACGTACTCCAGGAGAGTCGAGAAATTGTTCATTCCTTAATGCAATCTGTTTCCTTCTCTCTGATCCTCATTTTGCAGATAAATAAAGCTAAAGCCACTGAGTTTTCCCAGGTCACTTTTAAATAAGCCAGTCTCTCTGAGAG ATAGATGCCTGCCAAGGTGTAAGAACGT

### hs52.1

TACCCAGTAAATTGAGCATTACTCTAGGATTCTGAGACAGAGAGAAAGCACAATTTTAAAAGCTTTGCAGAGTTCCTTTGTAATTAGTCGCAGCTTTCCTTGAATATTAATTTTCCCTGCATCCCTTTCAAGTGGTTGAGAGACTGTCTCTACAACTACAGAGATGCACCCTCAGAACAACGACAGCAAGCGGCATGGTCTCAAACACCTATGATTA

### hs52.2

CAGAACGCACCCTCTGTTCTTCAGTGCAGTGTGTAGCTTATCAGTGCAAACAGTTTAATATTTATGCTAAGAGGATTGTCAAAAGCAGCTTCTGTTGCTTTAATTCTTGTTTTAAATAAATAATGAGAACATTTAAACACATTACTCTTCTTGGGGCCCCGGGGTCAGC TAATCTTATTATTTATGAAGTGATGTGCTACATAATAGTACTTAGTGCATGTTAACAGA

### hs52.3

CCTCTCTACCATCAGGCTTGGAAATGGGGCCAGGATATTCCATTCTTTGATCTCTTCATAGTCAGTCCTACACAGTCAGAAGACAAATAGTGAGCATGACCACTTTTTAATTGATTTAGACAAAAATGGAGAGAAGGCGGGGGTGGAGGGAGGCACATGTGCAATGCTCCCAAGTGTCCTCATAGTGTTTGGTTTTGATCCACTCGTTTTCACTGCCACGTACTCCAGGAGAGT CGAGAAATTGTTCA

## Results

### Genome-wide TFBS prediction and validation in mammalian genomes

**Figure 1.**
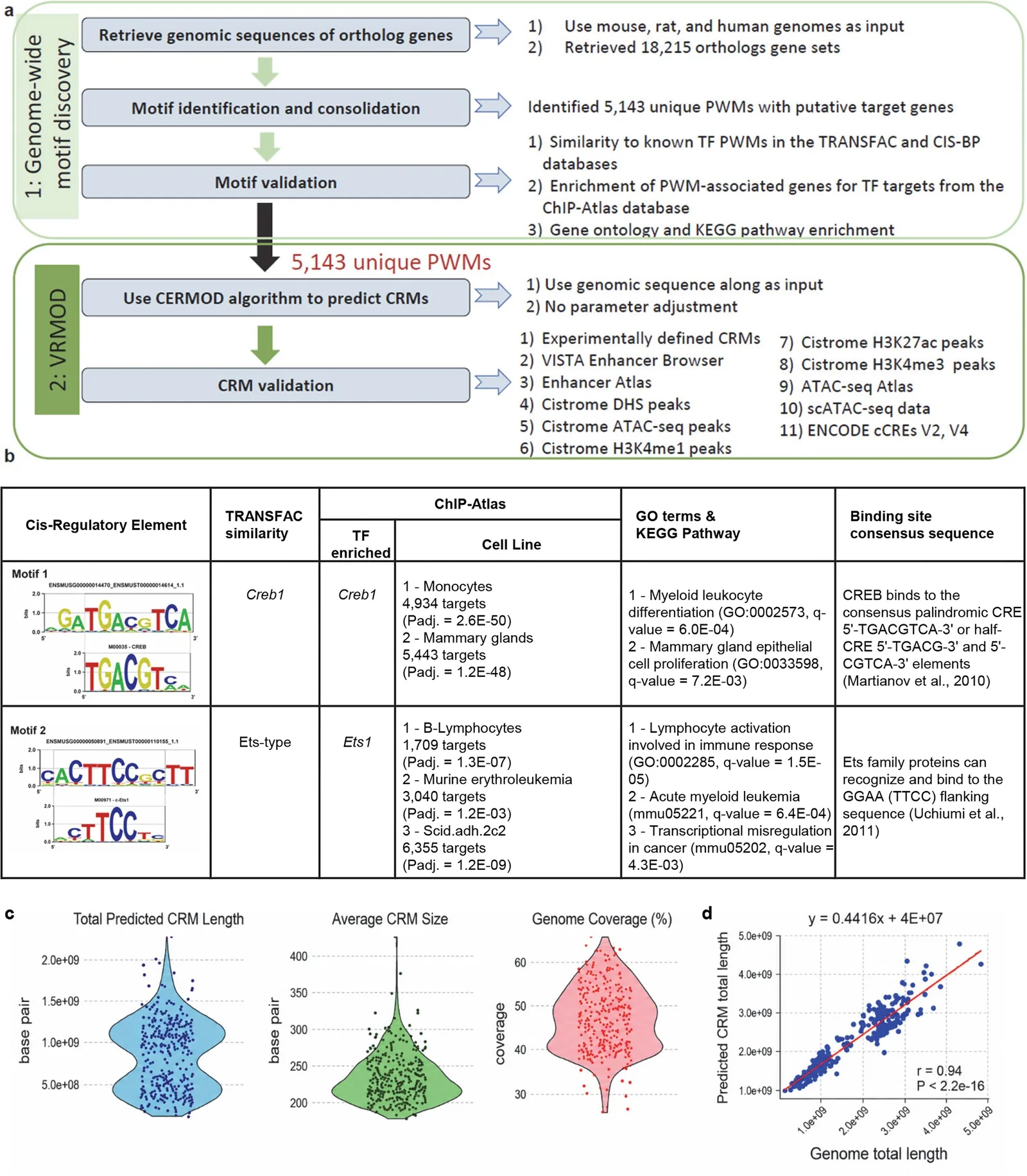
Whole-genome-wide CRM prediction and validation in mammalian genomes. (**a**) A schematic diagram of workflow of whole-genome-wide CRM prediction. In step 1, we defined a set of non-redundant conserved TFBS motifs in the mammalian genomes via applying PhyloNet on the genomic sequences of mouse, rat, and human. In step 2, we applied the algorithm implemented in CERMOD on the genomic sequences of human and mouse using the set of mammalian TFBS motifs obtained from step 1 to predict mammalian CRMs. (**b**) Examples of predicted PWMs and evidence supporting their functionality. For both Motif 1 and Motif 2, the top logos represent the predicted PWMs, while the logos below show the corresponding binding motifs of known transcription factors from the TRANSFAC database. Motif 1 (ENSMUSG00000014470_ENSMUST00000014614_1.1) corresponds to the TRANSFAC motif M00035 – CREB, whereas Motif 2 (ENSMUSG00000050891_ENSMUST00000110155_1.1) corresponds to M00971 – c-Ets1. (**c**) Violin plot of total predicted CRM length, average CRM size, and genome coverage distribution. (**d**) Correlation between genome length and total predicted CRMs length.

To understand the global transcriptional regulatory network of mammalian genomes, we first applied PhyloNet [61] to genomic sequences of humans, rats, and mice to identify a conserved network of TFBS motifs and their associated candidate target genes (Fig. 1a). PhyloNet systematically identifies phylogenetically conserved motifs that occur multiple times throughout the genome from whole-genome sequences of several related species, without relying on any additional information to define a network of regulatory sites for a given organism [61]. TFBS motifs were represented as PWM [72], and each matrix was associated with a set of putative target genes that contributed at least one conserved TFBS to the PWM, as determined by PhyloNet during the process of conserved regulatory motif identification. A final set of 5143 unique PWMs (*P*-value < 10^−10^) with an average length of 18 bp (ranging from 5 to 30 bp) was obtained after consolidation of redundant motifs as described in detail in the “Materials and methods” section [45]. Next, we performed multiple analyses to assess the functionality of the predicted PWMs. (i) We compared the predicted PWMs to known TF PWMs in the TRANSFAC [73] and CIS-BP databases [64] using the average log-likelihood ratio statistic [62] and OLAP score, as previously described [45] (Supplementary Table S1). Of the 5143 predicted PWMs, 836 (16.3%) and 2 203 (42.8%) shared similarities with at least one PWM from the TRANSFAC and CIS-BP databases, respectively. (ii) We tested whether each PWM-associated gene set was enriched for TF targets determined in the ChIP-Atlas database [65], which contains a collection of 2540 ChIP-seq datasets, including 723 mouse TFs in 369 different cell types and tissues. A total of 637 (12.4%) PWM-associated gene sets were significantly enriched for known TF targets under at least one condition (hypergeometric test, FDR-corrected *P*-value < .05). (iii) We tested whether the set of PWM-associated genes was enriched for specific classes of genes using GO and KEGG pathway enrichment analyses. We found that 2 678 (52%) PWM-associated gene sets were significantly enriched for at least one GO term, and 614 (12%) PWM-associated gene sets were significantly enriched for at least one KEGG pathway. We observed multiple lines of evidence from different categories consistent with the known functions of the corresponding TFs, which strongly supports that the majority of the PhyloNet-discovered PWMs represent DNA-binding specificity for the corresponding TFs (Fig. 1b and Supplementary Table S1).

### Genome-wide CRM prediction for 309 genomes across 500 million years of evolution

Previously, we published a CRM prediction algorithm that used a set of TFBS motifs derived from worm genomes to accurately predict CRMs in the *C. elegans* genome. It was named CERMOD [45]. To assess whether the algorithm implemented in CERMOD is applicable to mammalian genomes, we applied the same algorithm implementation [45] but used the set of 5143 mammalian PWMs obtained from the first step described earlier as the input (Fig. 1a) instead of the worm PWMs. We named it the VRMOD because, as shown below, the algorithm worked equally well on mammalian and other vertebrate genomes. The true test of any enhancer grammar model is its ability to identify enhancers in the genome from the sequence alone [8, 42–44]. In the CRM prediction step, VRMOD requires only genomic sequences as the input, without the need for any additional information. To test VRMOD, we applied it to 309 genomes downloaded from the Ensembl database (Supplementary Fig. S1). VRMOD has only one adjustable parameter, the motif occurrence enrichment *z*-score, and we used the same value consistently across all 309 genomes reported in this manuscript, namely a *z*-score ≥ 2.3263, which corresponded to a one-tailed *P*-value ≤ .01. VRMOD predicted a range of 489 733–6 856 502 total CRMs with an average length of 175–432 bp in each genome (Fig. 1c and Supplementary Table S2). The genomic coverage ranged from 26% to 65% with a strong correlation (Pearson’s correlation coefficient *r* = 0.94, *P* < .01) between the total length of the predicted CRMs and the genome size (Fig. 1c and d). Therefore, VRMOD-predicted CRMs represent a set of high-resolution genome-position-fixed CRM models, similar to position-fixed gene models for each genome.

### Comprehensive evaluation of VRMOD performance on human and mouse genomes

**Figure 2.**
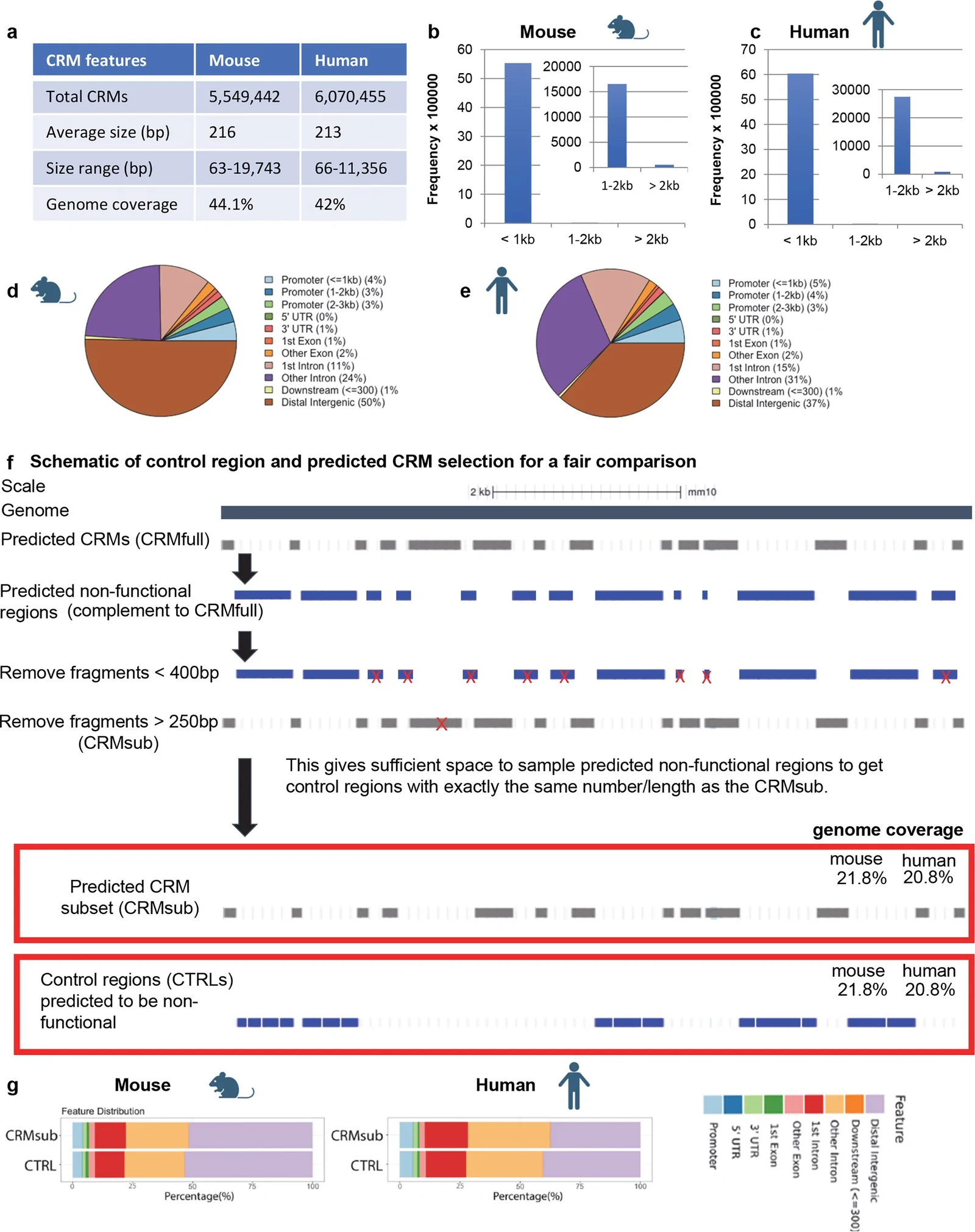
Human and mouse whole-genome-wide CRM prediction and method 1 for obtaining control regions for a fair evaluation of VRMOD performance. (**a**) Summary statistics of mouse and human complete set of predicted CRMs (CRMfull). (**b**) Mouse CRMfull length distribution. (**c**) Human CRMfull length distribution. The distributions of CRMfull relative to the genomic features for the mouse (**d**) and human (**e**) genomes, respectively. The length next to “Promoter” refers to the distance of the region from the transcription start site. (**f**) Schematic of control region and predicted CRM selection for a fair comparison. (**g**) Comparison of the distributions between CRMsub and CTRLs relative to the genomic features for the mouse and human genomes.

Because the human and mouse genomes are the best studied, with a vast amount of empirical data available, we first evaluated the VRMOD prediction performance for these two genomes. VRMOD predicted 5 549 442 (44.1% genome coverage) and 6 070 455 CRMs (42% genome coverage) with average sizes of 216 and 213 bp for the mouse and human genomes, respectively (Fig. 2a). The lengths ranged from 63 to 19 743 bp, but the majority (99.7% and 99.6% for mice and humans, respectively) were <1 kb (Fig. 2b and c). The majority of the predicted CRMs were located within distal intergenic regions or intronic regions (85% and 83% for the mouse and human genomes, respectively), with ~10% located within 3 kb of a promoter for both the human and mouse genomes (Fig. 2d and e), similar to that observed for the human putative regulatory elements defined by DHSs [20]. We refer to the full set of predicted CRMs for each genome as “CRMfull” herein for the mouse and human genomes.

#### Obtaining control regions for the evaluation of CRM predictions

To systematically evaluate the accuracy of the predicted CRMs, we need to obtain a set of control regions that do not have regulatory functions. However, there are few experimentally defined non-functional regions. Therefore, we devised a scheme to obtain a set of control regions with exactly the same number of DNA fragments, length distribution, and genomic coverage as a subset of the CRMs from each genome to ensure a fair and statistically robust comparison. These control regions were predicted to be non-functional in any tissue, cell type, or condition and were used as “negative controls” for evaluating VRMOD prediction performance, providing a stringent baseline for statistical comparison throughout our analyses (Fig. 2f, referred to as “method 1,” in the “Materials and methods” section). First, we obtained regions not covered by any predicted CRM, which corresponded to regions predicted to be nonfunctional. Because it is difficult to predict the exact boundaries of CRMs, small DNA fragments representing regions located between two closely located CRMs were removed, as they could be part of nearby functional CRMs. Due to insufficient non-functional sequence space to accommodate the full set of predicted CRMs (CRMfull), we created a length-restricted subset (referred to as the CRMsub with a length ≤250 bp) to enable the generation of properly matched control regions (referred to as CTRLs). This approach ensured matched comparisons, because CTRLs precisely mirror CRMsub in terms of the number of regions, length distribution, and genomic coverage. Through gradual elimination of the longest CRMs, we obtained a length-restricted predicted CRMsub such that we had sufficient space to generate a set of matched control regions within the predicted non-functional regions (CTRLs). As expected, the CRMsub and CTRLs had exactly the same number of DNA fragments, length distributions, genomic coverage, and similar positional distributions relative to the genomic features for each genome (Fig. 2g), with 21.8% and 20.8% genomic coverage for mice and humans, respectively. This approach provided statistically robust negative controls, while accounting for potential length-dependent effects or genomic distribution biases to enable a rigorous and fair evaluation of the predicted CRMs. CRMfull (all predicted CRMs) was used for genome-wide characterization, and CRMsub was used for OR calculations and statistical performance evaluations, as noted throughout the manuscript.

#### Evaluation of VRMOD performance using ExpCRMs curated for the human and mouse genomes

ExpCRMs still serve as the gold standard for functional CRMs. To evaluate the accuracy of the predicted CRMs, we performed an extensive literature search to identify mouse or human genes with genomic regions that have been analyzed using *in vitro* or *in vivo* reporter assays to locate any regulatory sequences. Wherever possible, we used regions that were identified using an enhancer assay with the minimal sequence required to drive reporter gene expression, because this better defines the boundaries of regulatory regions that are sufficient for regulation [45]. Only ExpCRMs <2.5 kb were included in the analyses because >90% of the peaks in the empirical datasets were <2.5 kb. The VISTA Enhancer Browser includes large DNA fragments that drive reporter gene expression in multiple tissues, suggesting the potential of including multiple enhancers. In mice, we curated 97 ExpCRMs associated with 56 genes, including enhancers, silencers, insulators, LCRs, and RNA post-transcriptional regulatory modules that regulate gene expression in diverse cell types and tissues at various developmental stages (Supplementary Table S3). The sizes ranged from 11 to 2000 bp, covering a total of 54 624 bp, with an average size of 563 bp. Similarly, 60 ExpCRMs associated with 43 human genes were collected, with 10 CRMs defined by mouse *in vivo* reporter assays and 50 by *in vitro* assays covering 20 610 bp, with an average size of 343 bp (range 25 to 1515 bp, Supplementary Table S4). An ExpCRM was deemed correctly predicted if the predicted CRM and ExpCRM overlapped at least 50% of the length of the smaller fragment or if they overlapped at least 1 bp (referred to as the 50%- or 1-bp-overlap cutoff hereafter).

##### Accurate prediction of enhancers and silencers at different genomic locations

We first evaluated VRMOD prediction accuracy using curated ExpCRMs that are enhancers or silencers located in the intergenic regions upstream of ATG start codons, the intronic sequences, or the 3′ untranslated regions (UTRs).

###### Accurate prediction of upstream CRMs

**Figure 3.**
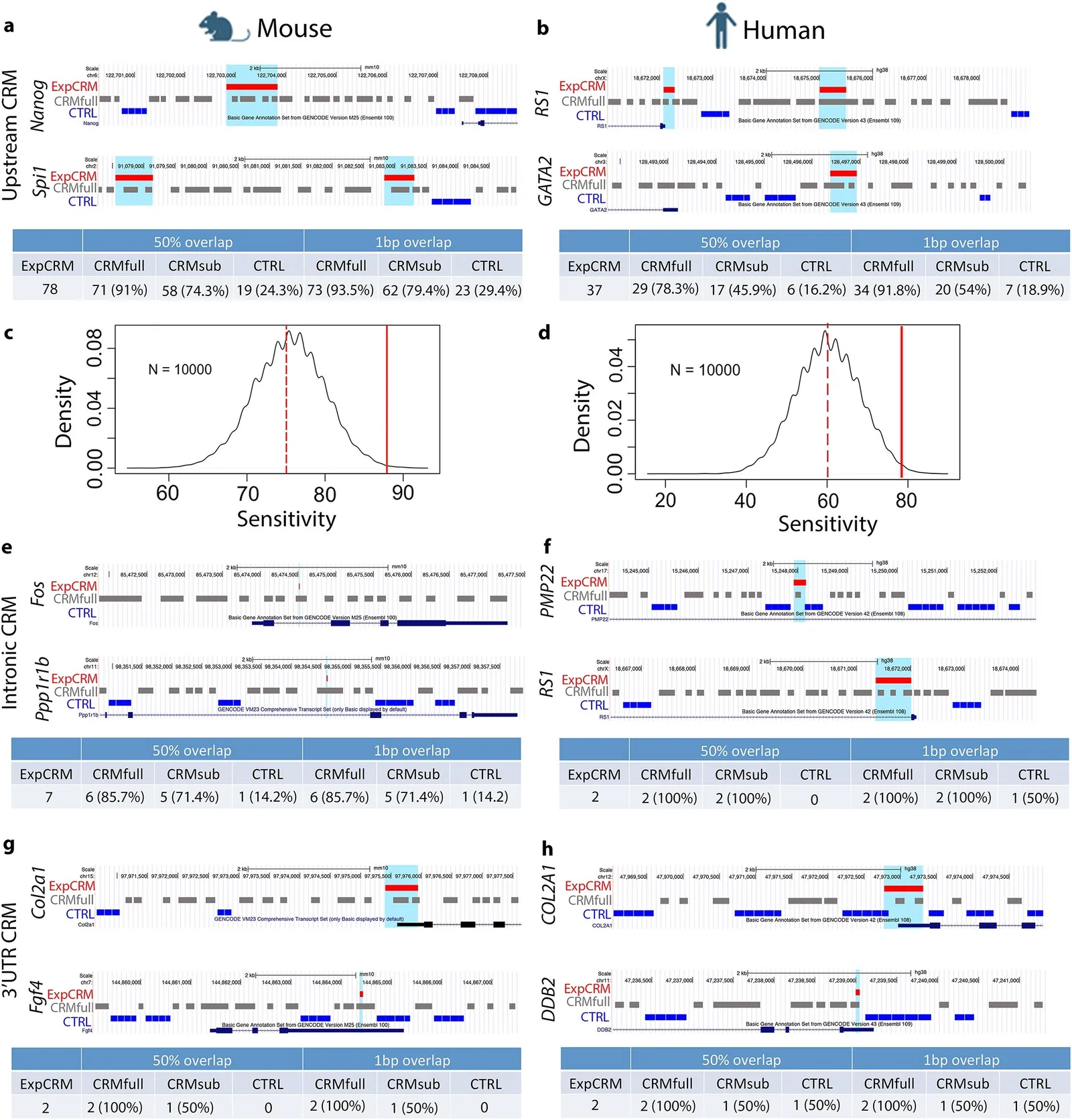
Comparison of predicted CRMs with experimentally defined modules located in the upstream intergenic regions, introns, and 3′ UTRs. Comparison between predicted CRMfull, CRMsub, and CTRLs with experimentally defined CRMs (ExpCRMs) located within 50 kb upstream of ATG start codon for (**a**) mouse *Nanog* and *Spi1*, and (**b**) human *RS1* and *GATA2*. (**c**) Comparison between the average sensitivity of simulated data (75.4%, 10 000 replications, dashed red line) and the actual sensitivity (88.4%, solid red line) for mouse data. (**d**) Comparison between the average sensitivity of simulated data (60.1%, 10 000 replications, dashed red line) and the actual sensitivity (78.3%, solid red line) for human data. Comparison between CRMfull, CRMsub, and CTRLs with ExpCRMs located in the intronic regions of (**e**) mouse c-*fos* and *Ppp1r1b* and (**f**) human *PMP22* and *RS1*. Comparison between CRMfull, CRMsub, and CTRLs with ExpCRMs located in the 3′ UTR of (**g**) mouse *Col2a1* and *Fgf4* and (**h**) human *COL2A1* and *DDB2*. Red bar: experimentally defined CRMs; gray bar: predicted CRMs; blue bar: CTRL regions; dark blue box and line: gene models.

We defined any modules that were located upstream of the ATG start codon and downstream of the 3′ UTR of the next nearest gene as upstream CRMs. We used ExpCRMs located within 50 kb upstream of ATG (69 mouse and 37 human ExpCRMs) and compared them to predicted CRMs located within the same genomic ranges. Figure 3a shows examples comparing the predicted CRMs with ExpCRMs located upstream of the *Nanog* homeobox gene (*Nanog*) and the Spi1 proto-oncogene (*Spi1*) in the mouse genome. *Nanog* enhancers drive gene expression in early mesoderm-specified progenitors [74]. *Spi1* has two CRMs previously reported in the literature, one driving reporter gene expression in transgenic mice at embryonic sites of the endothelial-to-hematopoietic transition and the other driving non-hematopoietic gene expression [75]. All three ExpCRMs were correctly predicted, without overlapping CTRL regions. We identified 78 mouse ExpCRMs upstream of the ATG. Seventy-one of the 78 ExpCRMs (91%) were correctly predicted, whereas only 19 (24.3%) overlapped with the CTRL regions at the 50%-overlap cutoff. Because CRMfull has higher genomic coverage than the CTRL regions, this comparison is not suitable for statistical testing. Therefore, simulations were performed by randomly shuffling the positions of the predicted CRMs within each sequence to estimate the statistical significance of a given sensitivity. Using mouse ExpCRMs within 50 kb upstream of ATG (69 ExpCRMs), the average sensitivity of the simulated CRMs was 75.4%, with a standard deviation of 4.4 for 10 000 simulations (Fig. 3c), demonstrating high sensitivity (88.4%, *P* < .01) for VRMOD predictions for mouse CRMs located within 50 kb of ATG (upstream regions). Similarly, all three enhancers upstream of human GATA-binding protein 2 (*GATA2*) [76] and retinoschisin 1 (*RS1*) [77] were correctly predicted without overlapping with the CTRL regions (Fig. 3b). A total of 29 (78.3%) of the 37 ExpCRMs overlapped with the CRMfull, whereas only six (16.2%) overlapped with the CTRL regions. The simulated CRMs had an average sensitivity of 60.1%, with a standard deviation of 7.7 (Fig. 3d; *P*-values < .01 for a sensitivity of 78.3%). We could not calculate the positive predictive value because the majority of the predicted modules were located within DNA sequences that have not been tested or because the DNA fragments drive reporter gene expression in additional tissues, but location information has not been reported [78].

###### Accurate prediction of intronic CRMs

The data presented in Fig. 3e demonstrate that both the mouse c-*fos* [79] and protein phosphatase 1 regulatory inhibitor subunit 1 B (*Ppp1r1b*) intronic enhancers (11 and 23 bp, respectively) [80] overlapped with the predicted CRMs, but not the CTRL regions. In total, six of seven (85.7%) intronic mouse ExpCRMs were correctly predicted, and only one overlapped with the CTRL regions. We collected two human intronic ExpCRMs, and both overlapped with the predicted CRMs, but not with the CTRL regions, at a 50%-overlap cutoff (Fig. 3f).

###### Accurate prediction of CRMs located in the 3′ UTR regions

We collected two mouse enhancers located in the 3′ UTRs of *Fgf4* (64 bp) [81] and *Col2a1* (543 bp) [82]. Both were correctly predicted without overlapping the CTRL regions at either cutoff (Fig. 3g). The two curated human ExpCRMs located in the 3′ UTRs of *COL2A1* (537 bp) [82] and *DDB2* (49 bp) [83] were also correctly predicted (Fig. 3h).

##### Accurate prediction of post-transcriptional CRMs

**Figure 4.**
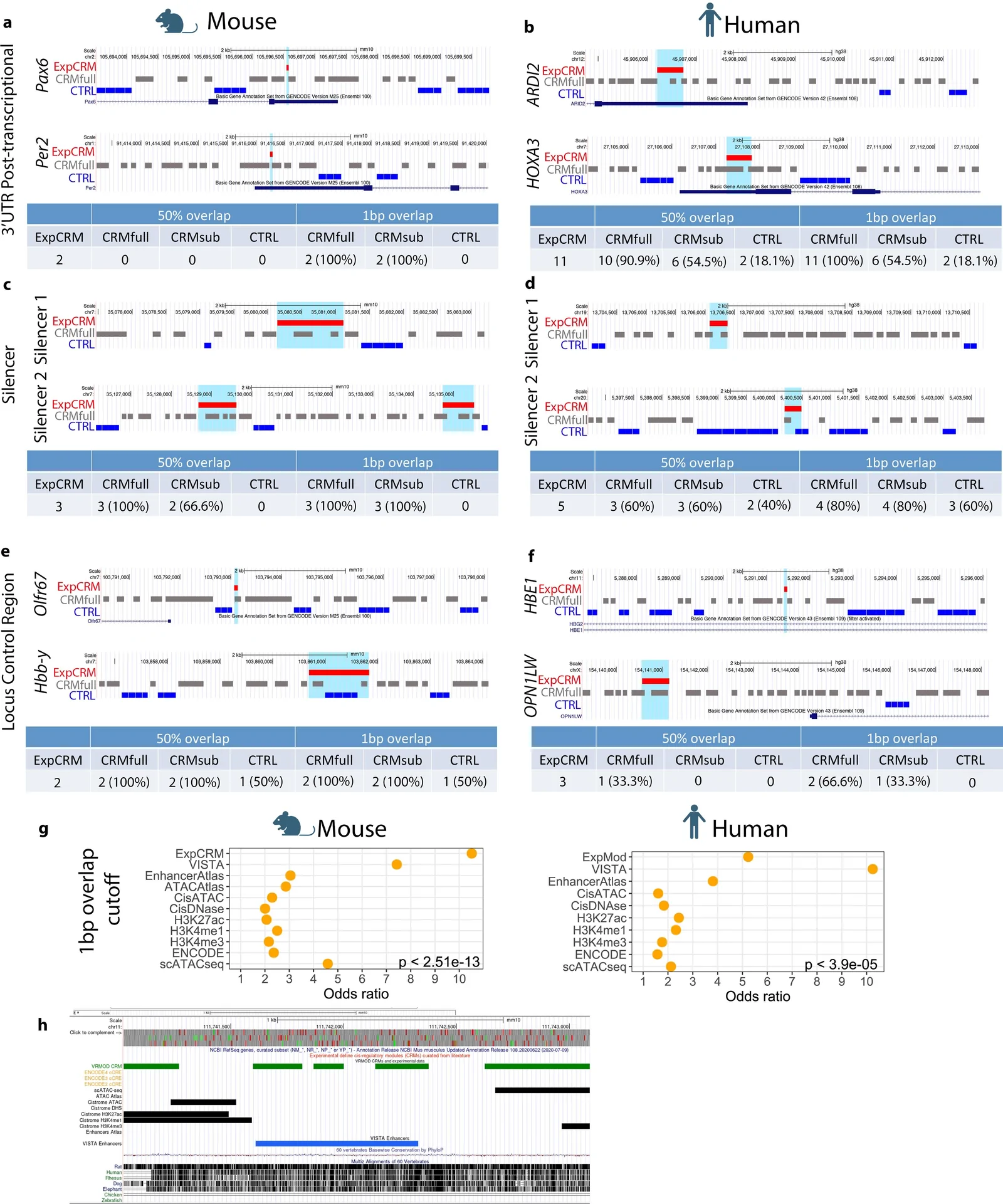
Comparison of predicted CRMs with experimentally defined modules (ExpCRMs) with different functionality and statistical evaluation of the whole-genome-wide CRM predictions. Comparison between CRMfull with the ExpCRMs with post-transcriptional regulatory function located in the 3′ UTRs of (**a**) mouse *Pax6* and *Per2* and (**b**) human *ARID2* and *HOXA3*. Comparison between CRMfull and predicted CRMs with silencer function for (**c**) the three mouse *Cebpa* silencers and (**d**) the two human silencer elements. Comparison between CRMfull with (**e**) mouse LCR of *Olfr67* (3′ HS1) and *Hbb-y* (5′HS2) and (**f**) human LCR of *HBE1* (5′ HS5) and *OPN1LW*. (**g**) Odds ratios and p-values comparing the sensitivity between CRMsub and CTRLs at 1bp-overlap cutoff. (**h**) mm2297 is an example of a VISTA Enhancer element that was classified as a “non-enhancer” at a e9.5, but was detected as a “positive element” at e11.5. mm2297 was predicted only by VRMOD, not by any version of ENCODE cCRE or experimental data. Blue bar: experimentally defined CRMs; green bar: predicted CRMs.

A 37 bp microRNA (miRNA) recognition element of *miR-375* in the mouse *Pax6* 3′ UTR was previously reported to be active in αTC1–6 cells [84], and a 43 bp *miR-24*/*miR-30a* binding site in the mouse *Per2* 3′ UTR was reported to function in NIH 3T3 cells [85]. Both post-transcriptional regulatory elements overlapped with the predicted CRMs at the 1-bp-overlap cutoff, but not with the CTRL regions (Fig. 4a). We collected 11 human elements validated functionally using a luciferase reporter assay [86]. All (100%) overlapped at least 1 bp with our predicted CRMs, and only two overlapped with the adjacent CTRL regions (Fig. 4b). These results demonstrated that our method identified post-transcriptional regulatory elements with high sensitivity and accuracy. Because miRNA sites are not included in the sequences used for PWM identification, our predicted CRMs may also represent novel transcriptional elements that happen to overlap with miRNA sites, as comparative genomic studies have suggested that many additional novel regulatory elements may exist in the mammalian 3′ UTRs [86]. One possibility is that they represent RNA-binding sites for TFs, because a recent study revealed that at least half of TFs also bind RNA, and TF-RNA interactions are a conserved feature important for vertebrate development and are disrupted in disease [87].

##### Accurate prediction of silencers

Silencer elements are under-characterized in mammalian genomes and can be difficult to predict. Three curated silencers (771–979 bp) that repress the activity of the *Cebpa* proximal promoter in mouse G1ME cells [88] were correctly predicted at both cutoffs, without overlapping with the CTRL regions (Fig. 4c). Of the five human silencers (301 bp) that were previously detected in K562 cells [89], VRMOD correctly predicted four (80%) at a 1-bp-overlap cutoff, with three of them also overlapping with adjacent CTRL regions (Fig. 4d).

##### Accurate prediction of locus control regions

Two mouse LCRs at β-globin loci [90] were both correctly predicted (Fig. 4e), although one also overlapped with nearby CTRL regions. We collected three human LCRs: two (both 72 bp) that demonstrated enhancer-blocking activity [90] and one upstream of *OPN1LW* (592 bp), which is essential for the expression of cone pigment genes [91]. Two were correctly predicted at the 1-bp-overlap cutoff, and none of them overlapped with the CTRL regions (Fig. 4f).

In summary, our predicted CRMs detected diverse classes of functional regulatory elements currently known in the human and mouse genomes with high sensitivity. Most ExpCRMs that overlapped with the predicted nonfunctional CTRL regions also overlapped with nearby predicted CRMs. For example, of the 22 mouse ExpCRMs that overlapped with the CTRL regions at a 50%-overlap cut-off, 19 (86.3%) overlapped with nearby predicted CRMs. This is largely because of the large size of some ExpCRMs, which may include both functional and non-functional sequences. Further experimental dissection is needed to identify the minimal functional sequences that will improve the accuracy of the performance evaluation.

#### Statistical evaluation of VRMOD predictions using multiple orthogonal datasets from the mouse and human genomes

VRMOD uses only genomic sequences for a given organism to perform predictions, without using any empirical data or phylogenetic conservation information at any step of CRM prediction. Therefore, the available empirical data can be used as orthogonal data to provide an objective evaluation of prediction accuracy. To perform statistical evaluation of VRMOD predictions, we used a compendium of experimentally defined enhancers and large-scale epigenomic datasets representing putative regulatory elements downloaded from multiple databases, including the VISTA Enhancer Browser [16], ENCODE cCREs V2 and V4 [50], the Enhancer Atlas 2.0 [49], the Cistrome Data Browser [51], ATAC-seq Atlas [70], and scATAC-seq data for the mouse brain and 45 adult/fetal human tissue types [52, 53] (Supplementary Tables S5 and S6). Experimentally defined enhancers were used as “gold standards” for CRM prediction evaluation. Peaks from empirical genomic and epigenomic datasets were used as CRM proxies, with the understanding that their imperfect ability to capture CRMs represents an inherent evaluation limitation. Peak overlap between each dataset and the CRMsub or CTRL regions was determined separately, and peak-detection sensitivity and ORs were calculated by comparing the CRMsub to CTRL. Following is a description of all datasets (12 for mice and 11 for humans) used in the analysis.

1. 157 literature-curated mice [97] and human [60] ExpCRMs driving gene expression in diverse tissues and cell types and at diverse developmental stages.
2. 1632 *in vivo* validated functional enhancer elements for mice (634) and humans (998) obtained from the VISTA Enhancer Browser.
3. 530 094 and 201 032 putative enhancers in mice and humans, respectively, annotated in 534 different tissue and cell types, obtained from Enhancer Atlas 2.0 [49].
4. DHS peaks from mouse (1 242 162 peaks) and human (2 401 388 peaks) DNase-seq data obtained from the Cistrome database. The DHS regions contain a variety of regulatory elements, including enhancers, silencers, insulators, and LCRs [50].
5. Bulk ATAC-seq peaks from the Cistrome database comprising 1 124 888 mouse ATAC-seq peaks derived from 1937 samples and 46 different tissue and cell types; human ATAC-seq data comprising 1 217 002 peaks from 1059 samples from 25 different tissue and cell types; and the Mouse ATAC-seq Atlas database [70] comprising 296 390 peaks derived from 66 ATAC-seq profiles of 20 primary tissues of adult mice.
6. ChIP-seq data for the histone marks H3K4me1 and H3K27ac, representing putative active enhancers, including ChIP-seq data for mouse H3K4me1 (1 095 988 peaks) and H3K27ac (1 400 986 peaks) and human H3K4me1 (1 565 782 peaks) and H3K27ac (1 697 483 peaks) downloaded from the Cistrome database.
7. ChIP-seq data for H3K4me3, which represent putative promoters, downloaded from the Cistrome database, with 1 120 149 peaks for mice and 2 081 430 peaks for humans.
8. scATAC-seq datasets, including 491 818 elements from 45 brain regions and 160 cell types in the adult mouse cerebrum from the mouse scATAC-seq brain atlas [52] and a human scATAC-seq dataset comprising ~1.2 million elements from 45 adult/fetal tissues and 222 cell types [53]. scATAC-seq detects cell type-specific open chromatin regions that potentially represent cell type-specific regulatory elements functioning in hundreds of cell types from different tissues and developmental stages.
9. Three versions of ENCODE cCREs, ENCODE2, ENCODE3, and ENCODE4, were generated through integrated analysis of ENCODE epigenomic datasets. ENCODE2 defined 368 121 and 1 063 878 cCREs across 169 mouse and 1518 human tissues or cell types, respectively [92]. ENCODE3 identified 339 815 elements in mouse and 926 535 in human genomes [50]. The ENCODE4 registry substantially expanded this catalog, comprising 926 843 mouse and 2 373 014 human cCREs [93]. ENCODE2 and ENCODE3 contained a comparable number of cCREs; however, discrepancies were observed in specific genomic regions. In some loci, ENCODE2 predicted more CRMs, whereas in others, ENCODE3 identified a greater number. In contrast, ENCODE4 represents a major expansion, with a 2.5-fold and 2.2-fold increase in cCREs relative to ENCODE2/3 for mouse and human genomes, respectively. Given the differences, we performed statistical evaluation using ENCODE2 and ENCODE4 datasets and provided visual comparisons of ENCODE2, ENCODE3, and ENCODE4 across genomic regions in the genome browser.

We first used all the collected ExpCRMs to evaluate the CRMsub and CTRL regions. For the 97 mouse ExpCRMs, CRMsub had a detection sensitivity of 79.3% (77/97), whereas the CTRL region had a detection sensitivity of 28.8% (26/97), resulting in an OR of 10.51 (*P* < 2.5e-13) at the 1-bp-overlap cutoff (Fig. 4g, Supplementary Table S7). At a more stringent 50%-overlap cut-off, fewer ExpCRMs were detected by CRMsub (71.1%) and CTRLs (22.6%), but the OR remained high (8.40, *P* < 1.5e-11, Fig. 4g, Supplementary Table S7). For the 60 human ExpCRMs, CRMsub had a detection sensitivity of 56.6% (34/60), whereas the CTRL region had a detection sensitivity of 20% (12/60), giving an OR of 5.23 (*P* < 3.9e-05, Fig. 4g, Supplementary Table S8) at a 1-bp-overlap cutoff. Using the more stringent 50%-overlap cutoff, we obtained similar results (OR = 4.67, *P* < 2.2e-04, Fig. 4g). When we used a 1-bp-overlap as the cutoff, the full set of predicted CRMs (CRMfull) had detection sensitivities of 93.8% (91/97) and 90% (54/60) for the mouse and human datasets, respectively (Supplementary Tables S7 and S8), even though CRMfull covered <45% of the genomes. Because of the large size of some ExpCRMs, they may include multiple CRMs and overlap with non-functional regions between two functional CRMs. For the 91 (93.8%) mouse ExpCRMs that overlapped at the 1-bp-overlap cutoff, 65 (67%) were exclusively located within the predicted CRMs, but none were located entirely within the CTRL regions. Similarly, of the 54 (90%) human ExpCRMs that overlapped at the 1-bp-overlap cutoff, 44 (81.5%) were exclusively located within the predicted CRMs, whereas only two were located exclusively within the CTRL regions. The vast majority of ExpCRMs that overlapped with the CTRL regions also overlapped with the VRMOD-predicted CRMs. Importantly, the percentage of ExpCRMs that overlapped within CTRL regions, but did not overlap any predicted CRMs, representing potential false negatives, was very rare, occurring in no cases in mice, and only 3.3% (2/60) of cases in the human genome. Lastly, predicted CRMs (CRMfull) overlapped with experimentally defined regions (ExpCRM) at frequencies significantly greater than expected by chance, as assessed by binomial tests. This enrichment was observed under both a 1-bp and a 50% overlap criterion in human (*P*-value = 3.21e-08 and 0.0156, respectively) and mouse (*P*-value = 2.55e-10 and 0.00346, respectively) genomes. Collectively, these results support the high sensitivity and accuracy of VRMOD predictions, while acknowledging that this statistical test has limitations, as the entire genome might not represent an optimal background for this test.

Next, functional enhancers from the VISTA Enhancer Browser database were used to evaluate the predicted CRMs. Although VISTA enhancers are also experimentally defined modules, they are much larger (average size of 2461 bp for the mouse genome) than our curated ExpCRMs (541 bp) (Supplementary Table S5). Many VISTA enhancers drive reporter gene expression in multiple tissues or brain regions without cell-type specificity information available. Therefore, DNA fragments in the VISTA enhancer database likely contain multiple enhancers, as well as non-functional sequences between these enhancers, which could be delineated through a more detailed analysis. With these limitations, a high percentage of VISTA enhancers overlapped with CTRL regions (68.5% and 61.2% at the 50%-overlap cutoff and 71.9% and 62.6% at the 1-bp-overlap cutoff for mice and humans, respectively). However, a much higher percentage of them overlapped with the CRMsub (92.8% and 92.2% at the 50%-overlap cutoff and 95% and 94.5% at the 1-bp-overlap cutoff for mice and humans, respectively), resulting in high ORs for all comparisons (5.94–10.24, *P* < 3.7e-15, Fig. 4g, Supplementary Tables S7 and S8). When we used the 1-bp-overlap cutoff for correct prediction, our full prediction (CRMfull) had sensitivities of 100% and 99.5% for the mouse and human datasets, respectively. Again, these results demonstrated the highly sensitive and accurate CRM predictions made by VRMOD.

Finally, we used multiple epigenomic profiling datasets to evaluate the predicted CRMs. For the mouse genome, we included 10 datasets containing 296 390–1 400 986 peaks per dataset, with an average size ranging from 270 to 947 bp (Supplementary Table S5). When comparing the overlaps between the collected peaks and our mouse predicted CRMsub and CTRL regions, CRMsub consistently had a markedly higher sensitivity (range 38.5%–82.3%) than the CTRL regions (range 31%–50.3%), with ORs of 1.28–4.58 (*P* < 2.2e-16) at either cutoff (Fig. 4g; Supplementary Table S7), highlighting the significant enrichment of functional elements in our predicted CRMsub compared to the CTRL regions. Similar results were obtained using all of the epigenomic datasets to evaluate human-predicted CRMs. We included 9 human datasets containing 201 032–2 401 388 total peaks per dataset, with an average size ranging from 268 to 952 bp (Supplementary Table S6). Our human-predicted CRMsub had a much higher sensitivity (range, 35.3%–74.2%) than the CTRL regions (range, 35.1%–49.8%) at either cut-off. All ORs were >1.21 (Fig. 4g; Supplementary Table S8) except for the ENCODE2 and ENCODE4 cCREs datasets, which had an OR of 0.97 and 1 at the 50%-overlap cutoff, but a much higher OR (1.57 and 1.65) at the 1-bp-overlap cutoff. This is likely because of the high resolution of predicted ENCODE cCREs (average length of 268–276 bp for mice and human cCREs across both versions) and our predicted CRMs (216 bp for mouse and 213 bp for human), which were difficult to overlap ≥50%, but can overlap at 1 bp.

To rigorously evaluate the prediction performance of VRMOD, we implemented an additional evaluation using an alternative approach for defining control regions (Supplementary Fig. S2a–c, referred to as “method 2”). This alternative method enabled us to (i) assess the consistency of prediction performance across different control sets and (ii) confirm the reliability of our sensitivity estimates through orthogonal validation strategies. In this method, we used a similar approach to obtain CRMsub and CTRL; however, CTRLs were not trimmed to obtain the same genome coverage as CRMsub. We excluded fragments with increasing length until a length cutoff was found, which led to subsets of predicted CRMs that had similar genomic coverage as the control regions (300 and 350 bp were used for mice and humans, respectively). The CRMsubs covered 26.4% and 28.2% of the mouse and human genomes, respectively (Supplementary Fig. S2a). The CTRL regions predicted to be nonfunctional covered 27.3% and 29.4% of the mouse and human genomes, respectively. Therefore, the control regions (predicted nonfunctional regions) were longer and had slightly higher genomic coverage than the predicted CRMsub, giving them an advantage in comparison. We obtained results similar to those described earlier, demonstrating high prediction accuracy and consistent performance (Supplementary Fig. S2d and e; Supplementary Tables S9 and S10). Notably, our prediction had higher ORs (5.91–16.32) compared to those of control regions when evaluated using curated ExpCRMs and VISTA enhancers, the “gold-standard” functional regulatory sequences, no matter what cutoff or control regions were used. To further evaluate the specificity of our approach, we generated an alternative negative control set derived from the ENCODE v3 cCRE catalog. We downloaded the ENCODE v3 cCRE annotations and used bedtools complement to identify genomic regions not overlapping any annotated cCRE. These complementary regions were divided into 200-bp windows, yielding 14 682 670 candidate negative fragments, from which we randomly sampled 4 490 452 fragments to match the size of our original predicted control (CTRL) set. We then cross-referenced this ENCODE-derived negative set against our original CTRL regions and our gold-standard collection of 60 experimentally validated human CRMs (ExpCRMs). The ENCODE-derived negative set overlapped with 41.54% of our original CTRL regions, indicating that the two approaches capture partially distinct genomic spaces. Notably, 45% of the ExpCRMs overlapped with the ENCODE-derived negative set, compared with only 20% overlapping our original CTRL regions, and 48% of VRMOD-predicted CRMs overlapped with the ENCODE-derived negative set. The similarity between the ExpCRM (45%) and VRMOD-predicted CRM (48%) overlap rates suggests that a substantial fraction of ENCODE cCRE-complement regions may still represent bona fide regulatory elements not yet annotated by ENCODE, rather than a definitive negative set. To directly test whether these non-cCRE predictions represent genuine regulatory elements rather than false positives, we asked what fraction of the ExpCRMs overlapping the ENCODE-derived negative set are also predicted by VRMOD. Of the 27 ExpCRMs (45%) that fell within non-cCREs, 19 (70%) were also predicted as CRMs by VRMOD, indicating that the large majority of experimentally validated CRMs missing from the ENCODE cCRE catalog are nonetheless recovered by VRMOD (Fig. 4h) In summary, the consistent prediction sensitivity and ORs when comparing CRMsub with CTRL regions for both mouse and human genomes across all datasets, together with the 70% recovery of non-cCRE ExpCRMs by VRMOD, representing diverse regulatory elements from thousands of samples derived from hundreds of different tissues and cell types at various developmental stages and conditions, suggested that our predicted CRMs comprehensively captured all types of regulatory elements tested here, unrestricted by tissue, cell type, developmental stage, or stimulus.

#### Evaluation of VRMOD performance on less-characterized genomes

The U.S. cattle industry, the largest agricultural sector, generated more than $86 billion in 2022 [94]. Although the sequence of the bovine reference genome has been publicly available since 2009, the *cis-*regulatory landscape remains largely unknown, resulting in limitations for exploiting the genome-to-phenome relationships [95]. Meanwhile, the U.S. poultry industry, the world’s largest [96], produced $76.9 billion in 2022 [96]. Chickens diverged from humans at least 500 million years ago [48], and their regulatory elements were not reported until recently [97]. Therefore, we manually curated experimentally defined modules for the bovine (*Bos taurus*, ARS-UCD 1.2) and chicken (*Gallus gallus*, GRCg6a) genomes and used them as gold standards to evaluate the prediction performance of VRMOD.

**Figure 5.**
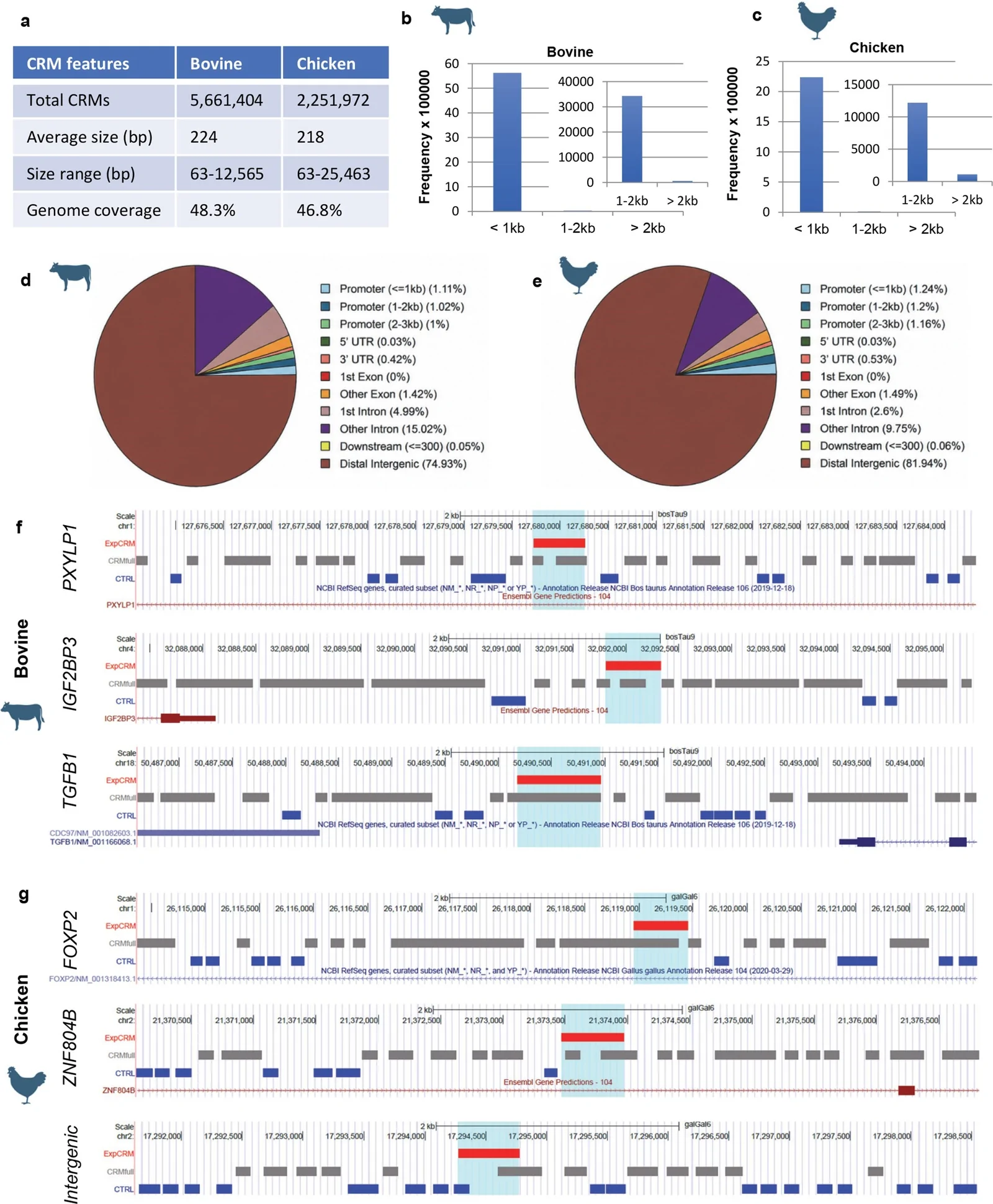
Comparison of bovine and chicken predicted CRMs with experimentally defined modules (ExpCRMs). (**a**) Summary statistics of bovine and chicken complete set of predicted CRMs (CRMfull). (**b**) Bovine CRMfull length distribution. (**c**) Chicken CRMfull length distribution. The distributions of CRMfull relative to the genomic features for the bovine (**d**) and chicken (**e**) genome, respectively. (**f**) Comparison of bovine CRMfull with three ExpCRMs located at different genomic locations close to *PXYLP1, IGF2BP3*, or *TGFB1*. (**g**) Comparison of chicken CRMfull with three ExpCRMs located in different genomic locations near *FOXP2, ZNF804B*, or in an intergenic region. Red bar: experimentally defined CRMs; gray bar: predicted CRMs. Blue bar: CTRL regions; blue box and red line: gene models.

VRMOD predicted 5 661 404 CRMs (48.1% bovine genome coverage) with an average size of 224 bp (Fig. 5a). The lengths ranged from 63 to 12 565 bp, but the majority were <1 kb (99.3%) (Fig. 5b). The majority of the predicted CRMs were located within the distal intergenic or intronic regions (Fig. 5d). These CRM characteristics are similar to those of the human and mouse genomes. To evaluate the sensitivity of detecting bovine CRMs, we collected 11 ExpCRMs (392–1052 bp) with enhancer activity in cattle myoblasts, which were validated using luciferase reporter assays [55]. All enhancers were correctly predicted at a 1-bp-overlap cutoff and 10 (90.9%) were correctly predicted at a 50%-overlap cutoff (Fig. 5f and Supplementary Fig. S3). We created CTRL regions using method 1 for the bovine genome, which represented the predicted non-functional regions. Only four out of 11 ExpCRMs overlapped with adjacent CTRL regions at the 1-bp-overlap cutoff (Supplementary Fig. S3b and c), and only two overlapped at the more stringent 50%-overlap cutoff (Supplementary Fig. S3c). Seven ExpCRMs (63.6%) were located exclusively within the predicted CRMs. However, none of the ExpCRMs were exclusively located within the CTRL regions, further supporting the hypothesis that the predicted CRMs were enriched for true functional regulatory sequences.

VRMOD predicted a total of 2 251 972 CRMs (46.8% chicken genome coverage) with an average size of 218 bp (Fig. 5a). The lengths ranged from 63 to 25 436 bp, but most (99.4%) were <1 kb (Fig. 5c). Approximately 82% of the predicted CRMs were annotated as distally intergenic (Fig. 5e). To evaluate the sensitivity of VRMOD for detecting chicken CRMs, we collected three published ExpCRMs (500 bp) that were tested using enhancer reporter assays *in vivo* in mouse brains [54]. The first enhancer was located in an intron of *FOXP2*, which drives GFP reporter gene expression specifically in the striatum and striatum-like amygdalar nucleus [54]. The *ZNF804B* enhancer drives reporter gene expression in the L2/3 IT and PIR neurons in the mouse brain [54], whereas the *KIAA1217* enhancer drives specific gene expression in the deep layers of the mouse neocortex, with strong activity in the hippocampus and thalamus [54]. The third enhancer is located in an intergenic region 183.6 kb upstream of the *KIAA1217* gene. All enhancers were correctly predicted at the 1-bp-overlap cutoff and two (66.6%) were correctly predicted at the 50%-overlap cutoff (Fig. 5g). Similarly, two ExpCRMs from the intronic regions of *Foxp2* and *ZNF804B* were exclusively located within the predicted CRMs (66.7%), whereas none of the ExpCRMs were exclusively located within the CTRL regions.

Next, we extended the genome-wide evaluation to diverse vertebrate organisms, including chicken (galGal6), pig (*Sus scrofa*, susSc11.1), rat (*Rattus norvegicus*, rn6), and zebrafish (*Danio rerio*, danRer11), using data available in the Enhancer Atlas database, as well as *C. elegans* (ce11), an invertebrate for which the algorithm was initially designed. We obtained comparable results across species with similar genome coverage, average CRM size, and sensitivity, with the exception of zebrafish (Table 1). At the stringent 50%-overlap cutoff, the sensitivity ranged from 63.51 in zebrafish to 87.20% in humans (Table 1). At the more lenient 1-bp-overlap cutoff, the sensitivity increased across all species, ranging from 75.96% to 93.02%. Notably, VRMOD performed very well in *C. elegans*, predicting 213 076 CRMs with an average size of 245 bp. Although the predicted CRMs covered only 52.16% of the genome, they detected 90.1% of the predicted enhancers from the Enhancer Atlas at the 50%-overlap cutoff and achieved a sensitivity of 96.09% at the 1-bp cutoff. Given that mammalian PWMs were used for predicting *C. elegans* CRMs, several factors likely contribute to this high sensitivity, including conservation of TFs across species [98–100], the slow evolution of TF-binding specificity [101–103], cross-species similarity of TF repertories [104], and conservation of DNA-binding domain (DBD) families, whereby TFs with related DBDs tend to recognize similar DNA motifs [64, 105]. Taken together, these findings indicate that the high sensitivity observed in *C. elegans* reflects the evolutionary conservation of the DNA-binding logic underlying transcriptional regulation. By assessing regulatory potential based on the aggregate TF-binding repertoire rather than individual motifs, VRMOD enables robust CRM prediction across distantly related evolutionary lineages.

**Table 1.** Evaluation of VRMOD prediction sensitivity using Enhancer Atlas data for different species at two overlap thresholds

Species	Predicted CRM genome coverage	Total number of peaks	Overlap at 50% cutoff	Sensitivity at 50% cutoff (%)	Overlap at 1 bp cutoff	Sensitivity at 1 bp cutoff (%)
Human	41.96%	200 956	175 243	87.20	185 674	92.39
Mouse	44.09%	530 089	446 362	84.20	491 472	92.71
Rat	43.76%	173 556	144 100	83.02	152 802	88.04
Pig	39.73%	2925	2518	86.08	2704	92.44
Chicken	46.66%	212 429	181 748	85.55	197 618	93.02
Zebrafish	40.39%	185 356	117 664	63.51	140 807	75.96
*C. elegans*	52.16%	20 148	18 155	90.10	19 362	96.09

We performed further genome-wide evaluations using ATAC-seq data from the FAANG Consortium (https://data.faang.org/home) [106] for available vertebrate genomes, including bovine, goat (*Capra hircus*, ARS1), chicken, and pig genomes. We also included 639 394 scATAC-seq peaks derived from over 94 000 cells sampled across six developmental stages during zebrafish embryogenesis at 10–24 h post-fertilization [56]. We achieved comparable results across species, with similar genome coverage, average CRM size, and sensitivity (Table 2).

**Table 2.** Evaluation of VRMOD prediction sensitivity using ATAC-seq data from the FAANG Consortium and published scATAC-seq peaks for different species at two overlap thresholds

Species	Predicted CRM genome coverage	Total number of enhancers	Overlap at 50% cutoff	Sensitivity at 50% cutoff (%)	Overlap at 1 bp cutoff	Sensitivity at 1 bp cutoff (%)
Bovine	48.1%	101 404	85 253	84.07	91 682	90.41
Chicken	46.66%	115 139	70 874	61.55	87 784	76.24
Goat	47.59%	74 805	74 805	82.61	66 263	88.58
Pig	39.73%	149 333	107 459	71.95	119 080	79.74
Zebrafish	40.39%	639 394	380 887	59.57	506 592	79.23

#### Comparison of VRMOD performance with three state-of-the-art methods

Lastly, we compared the performance of VRMOD with three state-of-the-art enhancer prediction resources, including Ni *et al*. 2021 [107], EnhancerAtlas [49], and ENCODE3 cCREs [50], using functionally validated enhancers from the VISTA enhancer database as the evaluation dataset, with genomic coverage calculated on the human hg38 assembly (Supplementary Fig. S3d). Ni *et al*. predicted a largely cell-type-agnostic but more comprehensive map of CRMs in the human genome by integrating all available TF ChIP-seq datasets. VRMOD achieved comparable genome coverage (42%) and sensitivity (99.5%) to Ni *et al*. (43.47% coverage and 100% sensitivity at *P* ≤ .05) with substantially higher base-pair resolution (average length of 213 bp versus 981 bp). To further compare the genomic regions identified by each method, we intersected VRMOD-predicted CRMs with the CRMCs reported by Ni *et al*. (Predicted CRMCs of Homo sapiens, version 2, hg38) using bedtools intersect. Of the 6 070 455 CRMs predicted by VRMOD, 3 708 959 (61.1%) overlapped a CRMC reported by Ni *et al*. Conversely, of the 1 399 719 CRMCs reported by Ni *et al*., 1 248 434 (89.2%) overlapped a CRM predicted by VRMOD. This asymmetry reflects the substantially larger and more granular set of CRMs predicted by VRMOD compared with Ni *et al*., such that the large majority of Ni *et al*.’s CRMCs are recovered within VRMOD’s broader predictions, while only a subset of VRMOD’s more numerous calls are represented in the Ni *et al*. set. Compared with EnhancerAtlas, VRMOD exhibited lower genome coverage (42% versus 58.78%) but higher sensitivity (99.5% versus 94.01%) and higher CRM resolution (213 bp versus 680 bp). Direct comparison between VRMOD and ENCODE3 cCREs based on sensitivity alone is not informative because VRMOD predictions span a larger fraction of the genome while also achieving higher sensitivity. However, VRMOD provided comparable resolution to ENCODE cCREs (213 bp versus 273 bp) and recovered many experimentally validated CRMs that are missed by ENCODE cCREs, as described in the rest of the manuscript.

In summary, our comprehensive evaluation demonstrated that VRMOD maintains a robust predictive performance across a wide evolutionary spectrum. The tool performed consistently in well-characterized human and mouse genomes, in less-studied vertebrate species, and even distantly related invertebrates, such as *C. elegans*. Notably, the performance for zebrafish was lower than that for other species in the Enhancer Atlas. Furthermore, the performance for pigs and chickens was lower when using data from the FAANG Consortium than when using data from the Enhancer Atlas. These results demonstrated that the evaluation results also depend on the reference data, which are considered “putative enhancers” before validated using gold-standard experimental approaches [3, 25]. It is unclear whether the low performance for zebrafish was because of the highly divergent genome sequence or a lower quality of predicted enhancers from the Enhancer Atlas. The high performance of VRMOD for the distantly related invertebrate, *C. elegans*, suggested that sequence divergence may not be a contributing factor. The stable performance across a broad phylogenetic distance, validated against multiple empirical datasets from diverse genomic databases, highlights the versatility of VRMOD as a reliable CRM prediction tool, regardless of the annotation status or evolutionary position of the target organism. Our work demonstrated the utility of VRMOD for species with limited available experimental data, such as chickens, bovines, pigs, and goats, which could be a valuable resource for understanding and manipulating gene expression to improve economically important traits. These results highlight the applicability of VRMOD across different genomes with an estimated evolutionary distance of >500 million years [48] and underscore its potential to contribute to the broader field of genomics beyond vertebrates.

### Using VRMOD prediction to guide experimental design

**Figure 6.**
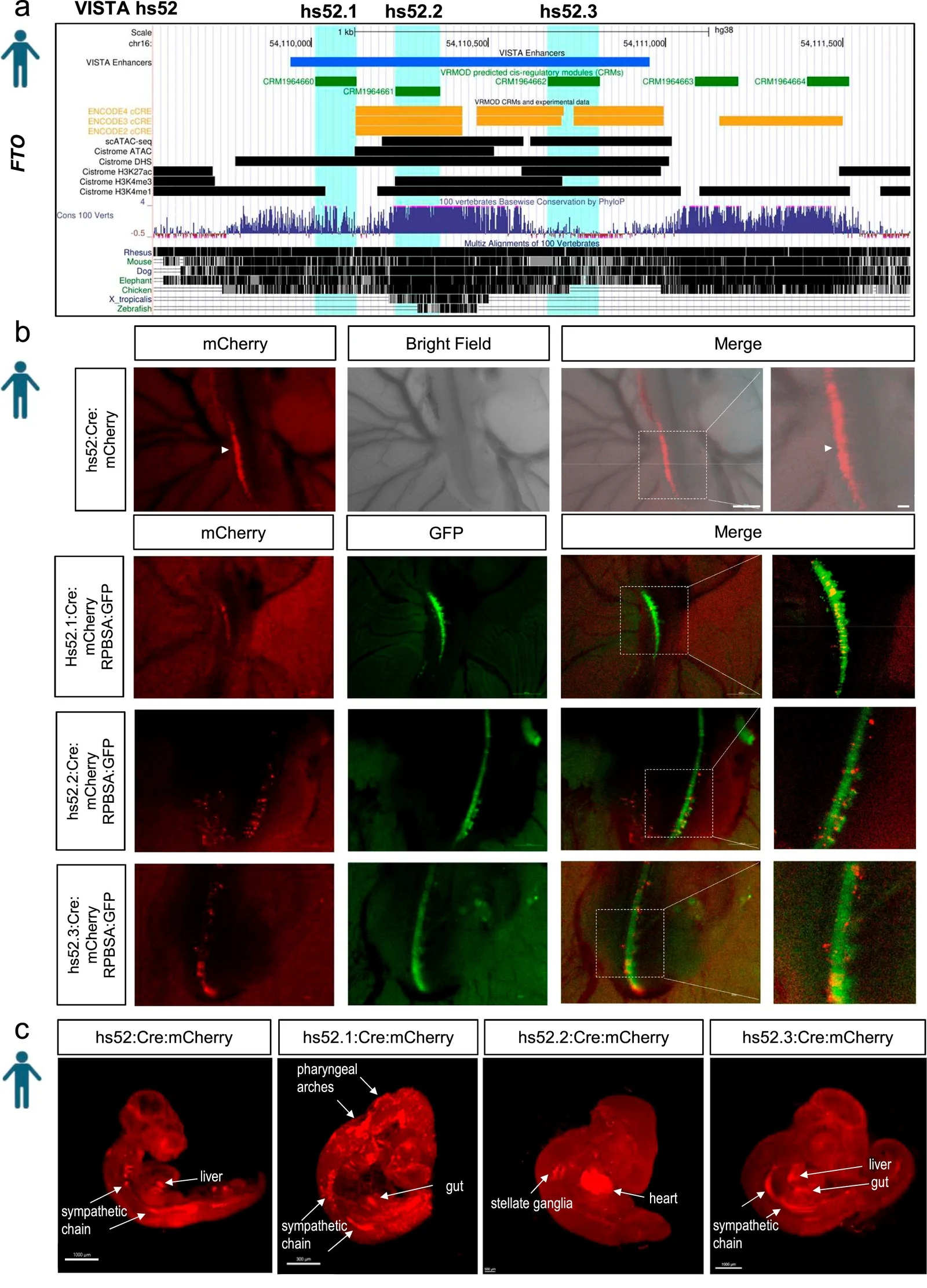
*In ovo* characterization of VISTA enhancer and predicted CRMs in chicken embryos via live imaging. (**a**) Schematic of the VISTA enhancer hs52 and the predicted CRMs on human chromosome 16. hs52.2 is highly conserved, whereas hs52.3 is highly diverged. (**b**) VISTA hs52, CRMs hs52.1, hs52.2, and hs52.3 enhancers driving mCherry expression in a whole-mount view in live chick embryo at 24 h after electroporation. RPBSA:GFP plasmid was co-electroporated. Arrowheads—neural tube. *n* = 5 independent animals per enhancer. Scale bars:1000 µm; zoomed image, 100 µm. (**c**) Whole embryo clearing 24 h after electroporation. *n* = 3 independent animals. Scale bar:1000 µm.

Many VISTA enhancers drive reporter gene expression in multiple tissues or brain regions, and therefore, likely contain multiple enhancers as well as nonfunctional sequences between these enhancers. To test this hypothesis, we used VRMOD-predicted CRMs to guide the experimental design to dissect large enhancers through a more detailed analysis, and tested their ability to drive reporter gene expression in chick embryos using *in ovo* electroporation [108, 109]. *In ovo* electroporation is a technique used to transfect cells of interest in living, developing chick embryos. This allows longitudinal observation of transgene expression in living embryos to obtain a more complete picture of the spatiotemporal expression of reporter genes. We were interested in the differentiation and migration of neural crest cells. These progenitor cells are located in the embryonic dorsal neural tube and differentiate into many derivatives, including peripheral nervous system components, such as sensory and autonomic neurons, satellite glia, Schwann cells, endocrine cells, and melanocytes [110, 111]. VISTA Enhancer hs52 (1013 bp) is located within an intron of the fat mass and obesity-associated (*FTO*) gene on human chromosome 16. *FTO* has been shown to be broadly expressed in >200 cell types or tissues, including the nervous system, lung, eye, blood, muscle, heart, liver, kidney, pancreas, thyroid gland, skin, intestine, lymph node, adrenal gland, spleen, bone marrow, stomach, gall bladder, and bone. hs52 was identified using transgenic mouse screens of highly conserved non-coding sequences in the human genome. It drives β-galactosidase reporter gene expression at embryonic day (E) 11.5 in the dorsal root ganglion and trigeminal ganglia in the mouse embryos. Three predicted CRMs overlapped with the hs52 enhancer: hs52.1 (116 bp), hs52.2 (127 bp), and hs52.3 (147 bp) (Fig. 6a). hs52 and the three VRMOD predicted CRM elements were amplified from human genomic DNA using polymerase chain reaction and cloned upstream of a Cre recombinase plasmid. To verify the specificity of the expression, enhancer::Cre plasmids were electroporated into stage 16–17 chick hemi-tubes along with a Cre-dependent mCherry plasmid (pCAGG-LoxP-STOP-LoxP-mCherry). GFP, driven by the general synthetic promoter RPBSA, was used as a control for electroporation efficiency (Fig. 6b). The hs52 element was widely expressed in many cells along the embryo neural tube, while the elements hs52.1, hs52.2, and hs52.3 showed more restricted expression in the dorsal view of live embryos at HH stage 23–24 (E4–E4.5) (Fig. 6b), although we cannot distinguish whether they are subpopulations of hs52+ cells. To further decipher the location of the cells driven by these elements, we performed whole-embryo tissue clearing to examine the tissue-specificity at HH stage 23–24 (E4–E4.5). hs52+ cells were distributed across various locations in the developing embryo, including multiple ganglia of the sympathetic chain and the liver, consistent with its expression in the peripheral ganglion in the mouse embryos assayed at E12.5. Interestingly, the three VRMOD-predicted CRMs all drove tissue/cell-type-specific expression. hs52.1+ cells were localized to multiple ganglia of the sympathetic chain, the gut region and the pharyngeal arches, including the trigeminal ganglion as seen for hs52 in the mouse embryos. hs52.2+ cells were localized to the developing heart and the stellate ganglia, a part of the sympathetic chain, whereas hs52.3+ cells showed expression along the sympathetic chain, the liver and the gut (Fig. 6c, Supplementary Videos 1–4). The observed expression patterns were consistent with the known tissue specificity of *FTO* transcripts, including the nervous system, heart, liver, and intestine, although whether hs52 directly regulates FTO remains to be determined. All three VRMOD-predicted CRMs drove reporter expression in the sympathetic chain, similar to hs52, but each also has its own unique tissue-specific expression, not necessarily a sub-pattern of hs52. Because mouse and chicken embryos were used to assess human DNA fragments, it remains uncertain which model better represents authentic human enhancer activity.

In summary, VRMOD-predicted CRMs are valuable for guiding experimental design to dissect larger enhancers and identify minimal sequences sufficient for driving tissue- and cell-type-specific gene expression. Of note, ENCODE2, ENCODE3, and ENCODE4 showed distinct predictions in this genomic region. CRM hs52.2 was consistently identified across all three versions, whereas hs52.3 was predicted only in ENCODE3 and ENCODE4. In contrast, CRM hs52.1 was not detected by any ENCODE cCREs version. Additionally, two VRMOD-predicted CRMs, CRM1964663 and CRM1964664, overlapped with an ENCODE3-predicted cCRE and a Cistrome H3K4me1 peak, supporting their potential regulatory activity. Interestingly, while CRM hs52.2 was highly conserved throughout evolution, with 93% nucleotide identity between the human and chicken genomes, the hs52.3 element had no detectable homology with the chicken genome (Fig. 6a and Supplementary Fig. S4a). The hs52.3 element is a fast-evolving element ranging from 93.55% nucleotide identity over its full length with *Macaca fascicularis* (macaque) to only 71.53–76.03% identity over 42%–58% of the query length for the pig, squirrel, and mole genomes (Fig. 6a and Supplementary Fig. S4b), with no statistically significant sequence similarity detected in the mouse or chicken genome. However, experimentally, both elements drive highly spatiotemporally regulated gene expression in chicken embryos, with at least partially overlapping tissue specificity with full-length hs52 in mouse embryos. Chickens and mice diverged from their human ancestors >500 million years and 85–96 million years ago, respectively.

### VRMOD prediction of non-conserved CRMs

**Figure 7.**
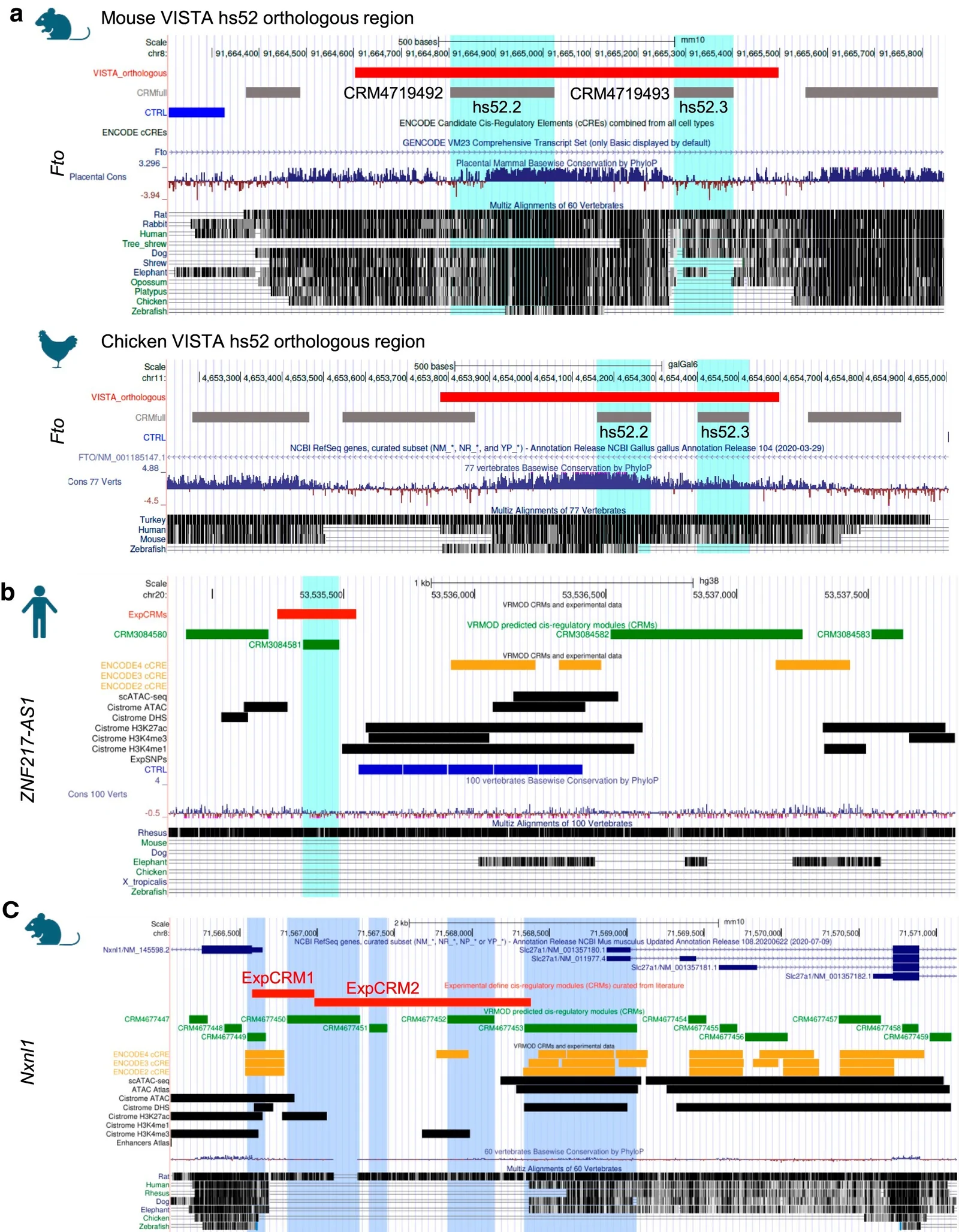
Prediction of non-conserved CRMs in mouse, chicken, and human genomes. (**a**) VISTA hs52 orthologous region of mouse and chicken showing that VRMOD independently predicted two CRMs in the mouse and chicken genome corresponding to the human hs52.2 and hs52.3 enhancers, respectively. (**b**) Example of human non-conserved ExpCRM located upstream *ZNF217-AS1* correctly predicted by VRMOD. (**c**) Example of mouse non-conserved ExpCRM located upstream of *Nxnl1* and *Slc27a1* correctly predicted by VRMOD.

Cross-species conservation has been used extensively in comparative genomics to identify regulatory sequences based on the assumption that functionally important genomic sequences are under more evolutionary constraints than sequences with less vital functions. However, a previous study from ENCODE showed that most regulatory regions are not constrained [112] and sequence conservation is poorly suited for discovering newly evolved regulatory modules or those that may have been gained or lost in a lineage-specific manner [32]. In addition, <5% of mammalian CRMs are conserved outside of eutherian mammals [112]. These factors render the identification of non-conserved CRMs challenging. The fact that VRMOD correctly predicted the hs52.3 element, a fast-evolving regulatory element, and that VRMOD does not require any additional information other than the query genomic sequence for CRM prediction inspired us to examine the capability of VRMOD to predict non-conserved CRMs. First, we identified the mouse and chicken orthologous regions of the human VISTA enhancer hs52 using the LiftOver tool [113] and checked whether any CRMs were predicted in that region. VRMOD independently predicted two CRMs (CRM4719492 and CRM4719493) corresponding to hs52.2 and hs52.3, respectively, which were located within the VISTA orthologous enhancer of the mouse and were similar to those of the chicken genome (Fig. 7a). Consistent with the human genome, the first CRM (corresponding to the hs52.2 element) was highly conserved, whereas the second CRM (corresponding to hs52.3) was highly divergent in both the mouse and chicken genomes. Therefore, VRMOD independently predicted orthologous enhancer regions using genomic sequences alone for each genome, without using any sequence conservation information.

Next, we examined whether human ExpCRMs that are not conserved in the mouse genome could be independently predicted by VRMOD. Using the LiftOver tool, we identified five human ExpCRMs that could not be mapped to the mouse genome (Fig. 7b and Supplementary Fig. S5a and b). VRMOD correctly predicted CRMs for all five cases (100% sensitivity) without using any sequence conservation information. For instance, a 301 bp silencer located upstream of human *ZNF217-AS1* was detected using an MPRA and was validated using a luciferase reporter assay [89] (Fig. 7b). VRMOD predicted CRM3084581, which overlaps with the human silencer in this non-conserved genomic region. Notably, this silencer was predicted only by VRMOD, not detected by any of the ENCODE cCRE versions or epigenomic datasets. Additionally, the *NCF1* enhancer was not detected by any of the ENCODE cCRE versions, whereas silencers of *YJU2B* and *LINC00658* were detected only by ENCODE4 (Fig. 7b and Supplementary Fig. S5a and b). Importantly, 22 out of 25 (88%) VRMOD-predicted CRMs were supported by at least one experimental dataset indicating regulatory activities, suggesting that these sequences represent bona fide functional regulatory sequences. Similarly, eight mouse ExpCRMs not conserved in the human genome were correctly predicted using VRMOD with 100% sensitivity (Fig. 7c and data not shown). The ~2 kb intergenic region between the start codon of *Nxnl1* and *Slc27a1* has two ExpCRMs. The 403 bp ExpCRM1 covers the conserved *Nxnl1* promoter, whereas the 1 399 bp ExpCRM2 represents a non-conserved negative regulatory element [114]. VRMOD predicted five CRMs in this region. CRM4677449 and the 3′-located ENCODE cCRE both overlapped with ExpCRM1. The other three predicted CRMs (CRM4677450, CRM4677451, and CRM4677452) overlapped with ExpCRM2. ENCODE did not identify cCREs within this experimentally defined functional region until the recent version of ENCODE4. Our predicted CRM4677453 sequence was consistent with the ENCODE cCREs located within the conserved promoter region of *Slc27a1* and supported by multiple lines of experimental evidence (Fig. 7c). All 13 VRMOD-predicted CRMs were supported by at least one experimental dataset, and many closely aligned with the boundaries of ENCODE cCREs.

Next, we evaluated whether sequence conservation affects CRM prediction sensitivity using human and mouse snATAC-seq peaks as an independent, experimentally derived benchmark of regulatory activity. Conservation scores for each peak were calculated using the GenomicScores package [71]. The peaks were then evenly divided into ten bins based on their conservation scores (Supplementary Fig. S6a). For the human genome, VRMOD demonstrated consistently higher sensitivity than CTRL regions across all bins for both CRMfull and CRMsub (Supplementary Fig. S6b). CRMfull consistently outperformed ENCODE2 but was less sensitive than ENCODE4 cCRE across all bins. For the mouse genome, VRMOD CRMfull and CRMsub demonstrated consistently higher sensitivity than CTRL regions as well as ENCODE2 and ENCODE4 across all bins (Supplementary Fig. S6c and d). These results support the conclusion that VRMOD can reliably predict CRMs, including those located in regions of low nucleotide-level conservation.

Next, we evaluated whether sequence GC content affects CRM prediction sensitivity using human, mouse, and zebrafish snATAC-seq peaks. For the human genome, we calculated the GC content for each peak and evenly divided the peaks into ten bins based on their GC content (Supplementary Fig. S7a). VRMOD CRMfull and CRMsub consistently achieved higher sensitivity than CTRL regions across nearly all bins, with exception of only two bins (bin 7, 8) where CRMsub showing comparable results (Supplementary Fig. S7b). VRMOD CRMfull and CRMsub also outperformed ENCODE2 cCRE in most bins. However, the recently released ENCODE4 had higher sensitivity across most bins than CRMfull. Notably, ENCODE2 and ENCODE4 cCREs sensitivity showed a strong positive correlation with GC content (Spearman correlation coefficient ρ = 0.927 and 1, respectively, *P* < .001), indicating substantial bias toward GC-rich regions. In contrast, VRMOD predictions (neither CRMfull nor CRMsub) showed significant association with GC content (*P* > .05), demonstrating that VRMOD predictions are robust to GC-content variation. For the mouse genome, VRMOD CRMfull and CRMsub consistently achieved higher sensitivity than CTRL regions, ENCODE2 and ENCODE4 cCREs across nearly all bins, except the bin with the highest GC content (Supplementary Fig. S7c and d). The sensitivity of CTRL regions (ρ = 0.73, *P* = .021), ENCODE2 (ρ = 1, *P* < .001) and ENCODE4 (ρ = 1, *P*-value < .001) cCREs showed a strong positive correlation with GC content, but not VRMOD predicted CRMs. For the zebrafish genome, we first generated control regions (CTRL) matched in number and length to a subset of predicted CRMs (CRMsub) in the zebrafish genome, using the same strategy applied to the mouse and human genomes. Zebrafish CRMsub and CTRL regions showed comparable distributions relative to genomic features (Supplementary Fig. S7e). Next, we calculated the GC content for each snATAC-seq peak and evenly divided the peaks into ten bins based on GC content (Supplementary Fig. S7f). Across low- to mid- GC content bins (bin 1–6), both VRMOD CRMfull and CRMsub consistently achieved higher sensitivity than CTRLs (Supplementary Fig. S7g), mirroring the pattern observed in the human and mouse genomes. For high GC-content bins, VRMOD CRMfull and CRMsub exhibited lower sensitivities than CTRLs. Notably, the sensitivity of CTRLs showed a strong positive correlation with GC content (ρ = 0.82, *P* < .01), whereas sensitivities of VRMOD CRMfull and CRMsub were significant negatively correlated with GC content (ρ = −0.82, −0.78, respectively; *P* < .05). ATAC-seq accessibility signals are well known to be positively associated with GC-content [115]; therefore, the elevated sensitivity of CTRLs in high-GC content bins is likely driven by GC bias inherent to ATAC-seq, rather than reflecting true regulatory enrichment.

In summary, VRMOD offers the distinct advantage of accurately predicting CRMs even within non-conserved genomic sequences, allowing the identification of regulatory elements that may be overlooked by conservation-based methods. The predicted CRMs of the 309 genomes will be a great extension of ENCODE cCREs resources to the research community.

### Identification of candidate causal variants associated with neurodegenerative diseases

Single nucleotide polymorphisms (SNPs) are linked to a wide range of human traits and diseases [116]. The detection of disease-associated SNPs has increased dramatically with the emergence of genome-wide association study (GWAS) technology [117–119], which has been used to detect >72 000 associations between genetic variants and complex traits and diseases [119]. Most GWAS-identified variants are located in non-coding regions of the genome, particularly within regulatory elements, indicating that many causal variants likely affect gene expression [116, 120]. However, a GWAS-reported SNP might not be the causal variant for a trait, because the reported tag SNP merely correlated with the causal SNP that was not measured in the study. To identify candidate causal SNPs and genomic regulatory sequences linked to neurodegenerative diseases, we integrated VRMOD predictions with empirical experimental data of regulatory sequences and multiple eQTL datasets associated with neurological diseases to prioritize SNPs and CRMs. eQTL analysis has identified genetic variants that influence gene expression levels [119, 121]. We collected 10 recently published eQTL datasets containing thousands of significant neurological-disease-associated SNPs from the following four sources: (i) the Religious Orders Study and Memory and Aging Project (ROSMAP) Study [122], a longitudinal clinical-pathologic cohort study of aging and AD; (ii) the Brain xQTLServe [123], which includes data from transcriptomic and epigenomic data for AD, schizophrenia, and bipolar disorder; (iii) the Online Neurodegenerative Trait Integrative Multi-Omics Explorer (ONTIME) [124], which includes various quantitative neurodegenerative disease endophenotypes for diseases such as AD, Parkinson’s disease (PD), frontotemporal dementia, amyotrophic lateral sclerosis, dementia with Lewy bodies, and multiple sclerosis; and (iv) MetaBrain, a large scale eQTL meta-analysis of previously published human brain eQTL datasets [125]. MetaBrain includes AD data from different brain regions and donors of different ancestries, such as African, European, and East Asian ancestries. Only the eQTLs that met the statistical significance threshold for the original study were included in our analyses.

**Figure 8.**
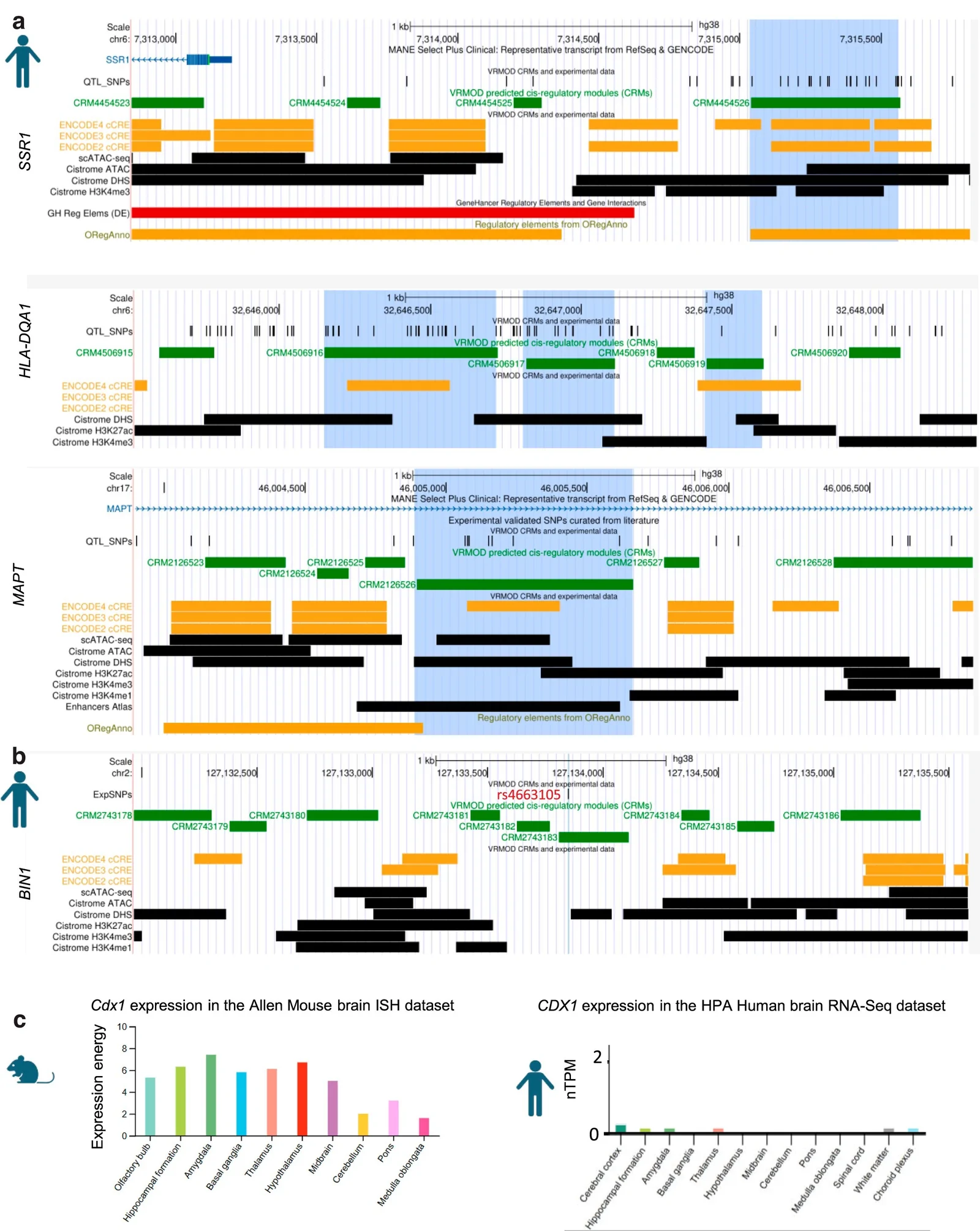
Prioritization of human predicted CRMs and identification of SNPs associated with neurological diseases. (**a**) Top CRM with the highest total number of lines of supporting evidence associated with the *SSR1, HLA-DQA1*, and *MAPT* genes. (**b**) Genomic view of rs4663105, an experimentally validated SNP (ExpSNP) linked to AD in the *BIN1* locus. Black bar: SNPs; green bar: epigenomic element; yellow bar: ENCODE cCREs (V2 and V3). (**c**) *Cdx1* expression in the brain from the Allen Institute and the HPA human brain RNA-seq database.

A total of 139–313 257 significant neurological-disease-associated SNPs were reported in the 10 datasets (Supplementary Table S11). We identified SNPs located within human-VRMOD-predicted CRMs, indicating their putative functional relevance to gene regulation. The percentage ranged from 34.5% to 45.8% for all datasets, which was similar to the 42% genomic coverage of the full VRMOD prediction. In total, 171 132 human-predicted CRMs contained one or more SNPs. Because CRMs function as gene expression regulatory units that can integrate the variant effects of multiple SNPs, we ranked the CRMs using a combination of the following two sources of evidence: (i) the total number of SNPs from all 10 eQTLs datasets and (ii) the number of lines of evidence supporting its regulatory function using experimentally defined modules, the VISTA enhancer database, and the genomic and epigenomic datasets described above (referred to as “regulatory evidence”). We summed the total number of lines of evidence (referred to as “disease-causal potential”) for each CRM and ranked them using this measurement. We then calculated the statistical enrichment of SNPs for each predicted CRM using the Phyper hypergeometric function. Of the 171 132 CRMs, 1884 were significantly enriched for SNPs with a FDR-adjusted *P*-value < .05 (Supplementary Table S12). CRM4454526 ranked first, with a total of 26 lines of evidence (22 eQTLs and four lines of regulatory evidence) and an FDR-adjusted *P*-value of 1.04e-40 (Fig. 8a). All SNPs that overlapped this CRM were obtained from the BrainQTL database and were associated with the signal sequence receptor subunit 1 (*SSR1*) locus on human chromosome 6. CRM4454526 is located 1.8 kb upstream of *SSR1*, which encodes a glycosylated endoplasmic reticulum membrane receptor and its expression level negatively correlates with dopaminergic neuronal survival in PD [126]. VRMOD track hub at the UCSC Genome Browser enabled the identification of additional evidence from the Genehancer database [127] and the Open the REGulatory ANNOtation database (ORegAnno) database [128] and all VRMOD-predicted CRMs had multiple lines of evidence supporting their regulatory activities. CRM4506916 ranked second, with 24 SNPs from BrainQTL and one line of regulatory evidence, with an FDR-adjusted *P*-value of 7.74e-45 (Fig. 8a). A nearby CRM (CRM4506917) had 12 SNPs from BrainQTL and one line of regulatory evidence with an FDR-adjusted *P*-value of 2.07e-20. The genes associated with these SNPs were three human leukocyte antigen (HLA) genes of major histocompatibility complex class II, namely, DR beta 1 (*HLA-DRB1*), DR beta 5 (*HLA-DRB5*), and DQ beta 1 (*HLA-DQB1*). The HLA locus plays an important role in the adaptive immune response and the HLA-DRB1*04 variant provides protection against AD and PD [129]. All VRMOD-predicted CRMs had at least one line of evidence supporting their regulatory activities. Two CRMs (CRM4506916 and CRM4506919) were not detected by earlier versions of ENCODE but were validated by ENCODE4 with closely aligned positions, demonstrating VRMOD prediction accuracy. Of note, 419 CRMs had SNPs associated with the *MAPT* gene on human chromosome 17, which encodes the microtubule-associated protein tau, the main component of the neurofibrillary tangle that is considered a central mediator of AD pathogenesis [130]. CRM2126526, located in the intronic region of *MAPT*, had 11 lines of evidence, including seven SNPs and four lines of regulatory evidence (adjusted *P* = 9.42e-06, Fig. 8a). It was noted that the three versions of ENCODE cCREs were different in this region. Neither ENCODE2 nor the ENCODE3 contained any of the *MAPT* SNPs covered by CRM2126526, which was detected only in the recent ENCODE4. Again, all VRMOD-predicted CRMs had at least one line of evidence supporting their regulatory activities and many of them closely aligned with ENCODE cCREs, demonstrating VRMOD prediction accuracy (Fig. 8a).

To validate our SNP prioritization results, we identified seven experimentally validated AD-associated non-coding SNPs (ExpSNPs) [131] for which sequence alterations led to changes in reporter gene expression levels (Supplementary Table S13). We found that three ExpSNPs overlapped with VRMOD-predicted CRMs. rs4663105 is associated with bridging integrator 1 (*BIN1*) on chromosome 2, which was detected only by VRMOD CRM2743183 (Fig. 8b). *BIN1 is* the second most significant risk locus associated with late-onset AD [132] and has been linked to amyloid pathology, tauopathy, and tau-dependent network hyperexcitability. This SNP is located 26.6 kb away from *BIN1* and disrupts the potential binding sites of four TFs: TEAD4, OSR2, CDX1, and VDR. Interestingly, *CDX1* is expressed in multiple brain regions in humans and mice (Fig. 8c) and regulates *BIN1* expression in pancreatic cells [133], supporting a potential causal relationship between *CDX1*, rs4663105, and disease pathogenesis in the human brain. The three versions of ENCODE cCRE differed significantly in this region, and none of them covered this SNP. Eight out of nine (88.9%) VRMOD-predicted CRMs had at least one line of evidence supporting their regulatory activities. rs11771145 overlapped with CRM5091149 and provided four lines of evidence supporting its potential regulatory function (Supplementary Fig. S8a). It is associated with the *EPHA1-AS1* locus on chromosome 7 and CRM5091149 is located in the intronic region of the gene. Interestingly, the three versions of ENCODE cCREs differ at this region with ENCODE2 and ENCODE4, but not ENCODE3, covered this SNP. CRM5091149 boundaries closely aligned with the boundaries of combined ENCODE cCREs at this position. rs3764650 is located in an intron of *ABCA7* on chromosome 19, which overlaps with CRM2373765 with seven lines of evidence (Supplementary Fig. S8b). VRMOD track hub at the UCSC Genome Browser enabled the identification of additional evidence from the Genehancer database for CRM5091149 and all VRMOD-predicted CRMs had multiple lines of evidence supporting their regulatory activities, further supporting the accuracy of VRMOD predictions.

In summary, our analysis identified single SNPs or clusters of SNPs in DNA fragments predicted by VRMOD to have regulatory functions, many of which had additional lines of evidence supporting their functionality. A comparison with experimentally validated AD-associated non-coding SNPs demonstrated the efficacy of our approach at identifying candidate AD-causal SNPs and revealing putative regulatory mechanisms. CRMs containing multiple neurological-disease-associated SNPs are likely to integrate the variant effects of co-occurring SNPs to regulate the expression of associated AD risk genes, which are great candidates for future mechanistic investigations.

## Discussion

CRMs are still poorly annotated, and their discovery is challenging [2, 25, 32, 134]. We developed the VRMOD method, which uses genomic sequences alone as the inputs to obtain accurate genome-wide CRM predictions. We applied VRMOD to 309 genomes currently available from the Ensembl database without adjusting any parameters. Extensive comparisons of predicted functional CRMs with predicted non-functional regions in well-studied human and mouse genomes and less-studied bovine, chicken, goat, pig, rat, and zebrafish genomes with curated ExpCRMs and large collections of genomic and epigenomic datasets representing putative regulatory sequences have demonstrated high sensitivity and accuracy. We demonstrated the value of VRMOD in helping the experimental dissection of a large VISTA enhancer to define smaller enhancers with more spatiotemporally restricted expression, identify non-conserved enhancers, and define candidate functional AD-associated non-coding SNPs. VRMOD public track hub at the UCSC Genome Browser enabled the visualization and integration of VRMOD-predicted CRMs with the 21 empirical datasets for the human and mouse genomes used in this manuscript as well as additional regulatory information from the Genehancer and ORegAnno database. Our VRMOD CRM prediction for the 309 genomes and the catalog of annotated functional neurological-disease-associated non-coding SNPs will have wide applications and broad impacts on research on transcriptional regulation in evolution, development, and disease pathogenesis.

How many regulatory elements are present in the mammalian genomes? Currently, there is a wide range of estimates of the number of regulatory elements in mammalian genomes, ranging from ~1.47 million CRMs, covering ~55% of the human genome [107] to predict saturation at ~4.1 million DHSs by ENCODE Project Consortium [92], which also reported that 80.4% of the human genome is covered by at least one ENCODE-identified functional element when considering all annotated elements. However, when RNA and broad histone elements are excluded, the coverage decreases to 44.2% of the genome, closely aligning with the VRMOD predicted CRM coverage of the human genome (42%). The ReMap 2022 database reported 2.4 million and 3.4 million non-redundant CRMs in their mouse and human regulatory atlases, respectively [135]. The Enhancer Atlas 2.0 database obtained >700 000 predicted enhancers across mouse and human species [49]. The accessible chromatin landscape of the human genome identified ~2.9 million DHSs through genome-wide profiling of 125 tissues/cell types [20]. The mouse scATAC-seq brain atlas identified 491 818 elements from 45 brain regions and 160 cell types in the adult mouse cerebrum alone [52]. Human scATAC-seq data have provided ~1.2 million elements from 45 adult/fetal tissues and 222 cell types [53]. Profiling additional tissues and cell types at additional developmental times and treatment conditions will certainly help identify additional regulatory elements. The recently released ENCODE4 cCRE includes 2.37 million human cCREs, covering 21% of the human genome and representing more than a twofold increase relative to ENCODE2/3 for both mouse and human genomes. Despite this expansion, a comparable proportion of ENCODE cCREs overlapped with VRMOD-predicted CRMs (77.2% in mouse and 72.4% in human for ENCODE4, compared with 79.5% in mouse and 72.2% in human for ENCODE2). Notably, VRMOD-predicted CRMs exhibited similar high resolution (average 213–216 bp) to ENCODE cCREs (average 268–276 bp), and many VRMOD CRM positions closely aligned with newly defined ENCODE4 cCREs. Collectively, these results provide strong evidence that VRMOD-predicted CRMs represent authentic regulatory sequences. Given the limitation of how many datasets are currently available, our prediction of ~5.5 million (44.1% genome coverage) and 6.1 million (42% genome coverage) CRMs for the mouse and human genomes, respectively, is a reasonable upper estimate of the regulatory universe of these genomes. Of note, for the 309 vertebrate genomes, even though the total number of CRMs varied greatly (489 733–6 856 502), most genome coverage was between 40% and 50%. Because the same prediction algorithm was used for genomes with evolutionary distances of >500 million years and it generated whole-genome-wide CRMs with similar characteristics in terms of length distribution, genomic location distribution, and genome coverage, and with similar prediction sensitivity, this suggests that the same organizing principle might be employed by all species to establish the regulatory network throughout evolution.

Our work provides a universal, common coordinate system and a stable reference for CRMs across 309 genomes, similar to the annotated genes for every coding gene in the genomes. This resource may revolutionize the analysis of epigenomic data. A crucial first step in analyzing epigenomic data, such as ChIP-seq, ATAC-seq, and DHS-seq data, involves identifying peaks that correspond to targeted DNA regions. Because no reference CRM models analogous to gene models exist, peak calling is required to define the genomic regions with the epigenomic signal of interest. Different peaks or peaks with different positions and lengths are commonly called in biological replicates and the number of peaks can vary greatly depending on the program used for peak calling [136]. This makes signal comparison across biological replicates and conditions much more challenging than comparing gene expression using the RNA-seq method, which uses a well-annotated set of gene models as a reference [137]. Many tools have been developed to define a consensus region from peaks overlapping among a set of replicates with varied exact positions; however, different tools yield very different consensus peaks with differing lengths and positions [137]. These differences are sufficient to influence downstream biological interpretations, and have led to disparate scientific conclusions regarding enhancer biology and disease mechanisms [138]. A similar issue is observed in ENCODE cCRE annotations, which show discrepancies across different versions, including variations in both element boundaries and the presence or absence of regulatory elements. VRMOD provides a comprehensive set of CRMs with fixed genomic coordinates across >300 genomes, analogous to reference gene models. This fixed-position feature enables consistent annotation and comparison across studies, unlike assay-derived peaks, which have boundaries that can vary depending on the experimental protocol or analysis pipeline. Similar to how transcription start sites of gene models have been precisely mapped, future studies may refine the CRM boundaries through experimental validation. These CRM models may serve as references for mapping reads directly to reference CRMs, similar to mapping reads to reference gene models in RNA-seq analysis. Therefore, data from different replicates and different conditions would have signal information within exactly the same genomic locations, enabling direct comparisons across samples and conditions, as well as the identification of differential CRM activities, similar to differentially expressed gene identification.

Our work greatly expands the experimentally defined CRM database and the regulatory universe for vertebrate genomes. It has been pointed out that the majority of putative elements identified by empirical methods are merely predictive, and biological validation is essential before assigning a definitive regulatory function to a genomic region [2]. Experimentally defined CRMs remain the gold standard of functional CRMs. However, because of the low-throughput, high-cost, and time-consuming nature of this validation approach, limited data are available. The VISTA Enhancer Browser is the only database with a collection of experimentally validated mammalian enhancers defined using *in vivo* reporter assays. Our literature-curated 157 experimentally defined CRMs were not available in VISTA, and expanded the current database by 9.6%. Despite the large number of available experimental datasets, we emphasize that we still do not have all the possible experimental data for every tissue, cell type, developmental stage, or stimulation condition. Therefore, our work fills a critical gap by providing a compendium for the base-pair resolution annotation of functional and non-functional elements in mammalian genomes. In addition, VRMOD can predict functional CRMs in non-conserved regions that are missed by ENCODE cCREs. Leveraging VRMOD predictions, we dissected a large human VISTA enhancer and discovered three previously unresolved sub-enhancers within hs52 at the FTO locus. Functional characterization in chicken embryos using 3D tissue imaging revealed that each sub-enhancer drives restricted spatiotemporal activity within a subset of the tissues regulated by the full enhancer. Because only genomic sequences are required as the input, without the need to change any parameter, VRMOD can be applied to any vertebrate genome, such as the planned reference genome assemblies of ~70 000 vertebrate species from the Vertebrate Genomes Project [139], hundreds of new genomes from the Bat1K Project [140] and the Zoonomia Consortium [141]. We expect to achieve the same prediction sensitivity and accuracy for any new vertebrate species or even invertebrate species.

This study provides valuable resources to the research community. First, because our computational prediction was independent of the empirical data used to evaluate the predicted CRMs, the combination of these two orthogonal types of information provided strong support for the functionality of a genomic region. Second, the small size (average of ~200 bp) of the predicted CRMs is ideal for MPRAs because most companies generally synthesize libraries of candidate enhancers of ~200 bp [142, 143]. Third, GWASs have identified many non-coding variants that are in linkage disequilibrium with the causal variant, but have not been able to pinpoint causal variants in general. Our SNP prioritization approach identified candidate functional non-coding neurodegenerative-disease-causal variants by integrating VRMOD predictions, eQTL, and empirical enhancer data. This approach can be applied to any disease or complex trait in any vertebrate species to elucidate the genetic underpinnings of diseases and complex traits. While several recent studies have integrated multiomic data for variant prioritization [144–146], VRMOD provides a unique framework that 1) identifies regulatory elements independent of existing tissue- or cell-type-specific data that may lack disease relevance, 2) pinpoints CRMs that potentially harbor actual functional variants associated with nearby tag SNPs, and 3) connects non-coding variants to specific TF-binding disruptions. Our identification of the CDX1-rs4663105 regulatory axis exemplifies how this approach can generate testable mechanistic hypotheses for GWASs. Finally, our work provides base-pair-resolution regulatory sequence annotations that greatly expand the current ENCODE cCREs registry, including non-conserved regulatory elements. Despite low sequence conservation, the successful prediction of orthologous CRMs in non-primate species (Fig. 7a) suggests exciting opportunities for comparative studies of regulatory evolution. Experimental characterization of these predicted elements across species will provide valuable insights into the evolution of gene regulation. VRMOD-predicted CRMs will enable the systematic evaluation of CRM sequences and the regulation of CRM activity during development, evolution, environmental stimulation, and disease pathogenesis for >300 species across >500 million years of evolutionary distance. The tissue/cell-type specificity of CRMs provides valuable insights into their phenotypic evolution and disease mechanisms. However, current datasets are limited to specific tissues, cell types, and developmental stages, with variable quality across collections. Existing SNP prioritization approaches rely on incomplete functional annotations and therefore may overlook causal variants that act in uncharacterized regulatory contexts. In contrast, the annotation-agnostic framework of VRMOD overcomes this limitation by comprehensively identifying candidate regulatory SNPs independent of existing datasets. Notably, VRMOD successfully identified the experimentally validated variant rs4663105 at the *BIN1* locus, which was missed by other methods, underscoring its improved sensitivity and predictive utility. Furthermore, because VRMOD predictions are orthogonal to experimental data, they can be complemented with tissue/cell-type specificity information from existing datasets. By integrating GWAS SNPs, disease-associated eQTLs, epigenomic datasets, and TF binding information with VRMOD predictions, we obtained a more complete regulatory landscape, including tissue/cell-type specificity, when supporting data were available. This framework becomes increasingly powerful as more empirical data become available, because our CRM predictions require no reprocessing to incorporate additional biological contexts. The regulatory sequences identified by both VRMOD prediction and experimental data represent the strongest candidates for disease relevance, potentially harboring true functional non-coding variants linked to nearby tag-SNPs within the same CRM.

The correlation analysis between VRMOD-predicted CRM length and genome size across 309 genomes provides the first systematic, whole-genome-scale evaluation of CRM sequence evolution spanning >500 million years of evolutionary distance. The observed relationship between genome size and CRM coverage is intriguing and not yet fully understood, largely due to limited knowledge of what proportion of any genome is composed of regulatory DNA and the incompleteness of genome assemblies. Nonetheless, several lines of evidence suggest that regulatory sequence content tends to positively correlate with genome size, which may help explain the observed tight correlation between the length of all the predicted CRMs and the genome size. First, protein-coding density decreases with genome size. Across eukaryotes, the proportions of protein-coding exons are inversely correlated with genome size (*r* = −0.6302, *P* < .0001) [147]. This implies that larger genomes allocate a greater fraction of their DNA to non-coding, and potentially regulatory, functions. Second, intronic content scales with genome size. Intron number and length increase with genome size across eukaryotes in general [147] and introns often harbor *cis-*regulatory sequences including abundant enhancers [148, 149]. Larger genomes, like those of vertebrates, have more introns per gene (e.g. humans have an average of eight), providing additional substrate for regulatory sequences. More introns per gene does not necessarily mean more *cis-*regulatory sequences per gene if a lower fraction of intron sequences act as *cis-*regulatory elements. Third, repeat and transposable element (TE) content, which comprise almost 50% of mammalian genomic sequence [150], are positively correlated with genome size [147]. TEs are well-established source of regulatory elements such as enhancers, promoters, insulators, and silencers [150]. Thus, larger genomes likely contain more TE-derived regulatory DNA, although it remains to be determined how much repeat and TEs contribute to the regulatory landscape and the correlation. Fourth, it has been shown that intergenic distance reflects regulatory complexity. In species such as *C. elegans* and *D. melanogaster*, intergenic distance is shaped by regulatory information contained in non-coding DNA [151]. Gene density directly reflects local regulatory complexity, such that the amount of non-coding DNA between a gene and its nearest neighbors correlates positively with that gene’s regulatory complexity. Genes with complex functions are flanked by significantly more non-coding DNA than genes with simple or housekeeping functions. Genes of low regulatory complexity are associated with approximately the same amount of non-coding DNA in *D. melanogaster* and *C. elegans*. This effect becomes more pronounced in larger, more complex genomes. With the availability of Telomere-to-Telomere complete genomes [152] and increasing accurate and comprehensive annotation of non-coding regulatory sequences, we will be better positioned to elucidate the relationship between regulatory sequence content and genome size.

VRMOD has several limitations in revealing the transcriptional regulatory mechanisms. First, it does not provide information about the tissue or cell-type specificity of CRMs, even though most CRMs are tissue- and cell-type-specific. However, our framework enables seamless integration with emerging single-cell datasets (e.g. scATAC-seq) to address this limitation and enhance the utility of VRMOD, particularly for candidate causal variant inference. Second, VRMOD predictions for all vertebrates were made using motifs conserved in the human, mouse, and rat genomes; however, there are TFs that are not shared across these species. This may have affected the performance of VRMOD in distantly related species. Third, there are no gold-standard negative datasets available for systematically evaluating the specificity of VRMOD (accuracy on non-CRMs), because CRMs in many cell types have not been experimentally assayed. ENCODE-complement regions are a useful but imperfect negative set, since a large fraction of them still overlap ExpCRMs/VRMOD predictions (likely reflecting cell-type-specific or developmentally restricted elements ENCODE hasn’t profiled). Fourth, many sequences other than TF motifs are involved in determining whether a sequence has *cis-*regulatory activity, including those involved in nucleosome positioning and DNA shape. Because VRMOD relies solely on regulatory motifs to define CRMs, this may lead to false-positive predictions. Lastly, for each predicted CRM, we sought to identify GC content-matched genomic regions of identical length to serve as control sequences and to directly compare their performance with the VRMOD-predicted CRMs. However, we encountered multiple practical challenges in identifying or adapting an existing computational tool capable of generating genome-wide GC-matched control regions while preserving variable CRM lengths on a per-element basis. This represents a limitation of the current evaluation. As an alternative, we assessed the relationship between VRMOD prediction and GC content and found that VRMOD predictions (both CRMfull and CRMsub) showed no significant association with GC content. These results indicate that VRMOD predictions are robust to GC-content variation, alleviating concerns that model performance is driven by GC bias rather than genuine regulatory signal.

## Supplementary Material

lqag107_Supplemental_Files

## Data Availability

VRMOD-predicted human CRMs, together with the genomic and epigenomic data used in the manuscript can be explored through the VRMOD public track hub hosted on the UCSC Genome Browser, which also provides data download information: Human genome: https://genome.ucsc.edu/s/gzhao/VRMOD_hg38. Mouse genome: https://genome.ucsc.edu/s/gzhao/VRMOD_mm10 The code and all relevant data have been submitted to the WashU Epigenome Browser and can be visualized at the following URLs: Human genome: https://epigenomegateway.wustl.edu/browser/?sessionFile=https://wangftp.wustl.edu/∼dli/gzhao/CRMs-202207/hg38-s.json Mouse genome: https://epigenomegateway.wustl.edu/browser/?sessionFile=https://wangftp.wustl.edu/∼dli/gzhao/CRMs-202207/mm10-s.json Here are the links for downloading the predicted CRMs for the 309 genomes: https://doi.org/10.5281/zenodo.15522503 https://doi.org/10.5281/zenodo.15531707 https://doi.org/10.5281/zenodo.15531746 https://doi.org/10.5281/zenodo.15531816 VRMOD source code, dependency documentation, setup instructions, and example test files for reproducing an analysis can be found at 10.5281/zenodo.21935162 and https://github.com/GuoyanZhao-Lab/VRMOD
