# Supplementary material for: Unlocking cis-regulatory landscapes across 500 million years of evolution and disease mechanisms
Source: NAR Genom Bioinform. 2026 Sep 15;8(3):lqag107. doi: 10.1093/nargab/lqag107 (PMC13575426; doi:10.1093/nargab/lqag107)
Supplement: Supplementary file 1 [file lqag107_supplemental_files.zip › Suppl_Figure1_Ensembl_genomes_2024_12_06_corrected.pdf]

Supplementary Figure 1

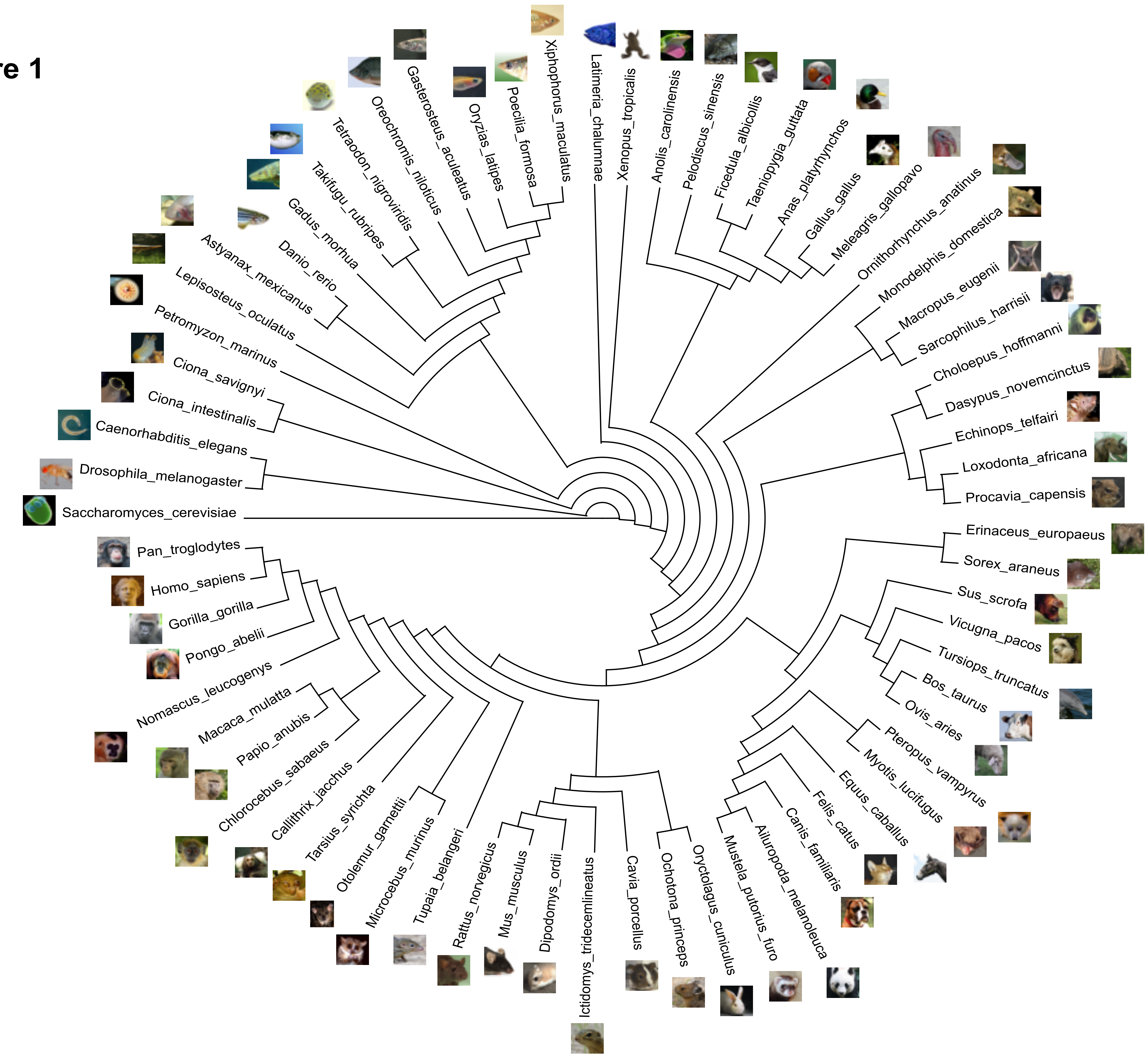

Image obtained using Dendroscope (D.H. Huson and C Scornavacca, Dendroscope 3: An interactive tool for rooted phylogenetic trees and networks, Systematic Biology, 2012)
