# Supplementary material for: Unlocking cis-regulatory landscapes across 500 million years of evolution and disease mechanisms
Source: NAR Genom Bioinform. 2026 Sep 15;8(3):lqag107. doi: 10.1093/nargab/lqag107 (PMC13575426; doi:10.1093/nargab/lqag107)
Supplement: Supplementary file 1 [file lqag107_supplemental_files.zip › Suppl_Figure3_VRMOD_Bovine_CRMs_2026_04_21_Submitted.pdf]

Supplementary Figure 3

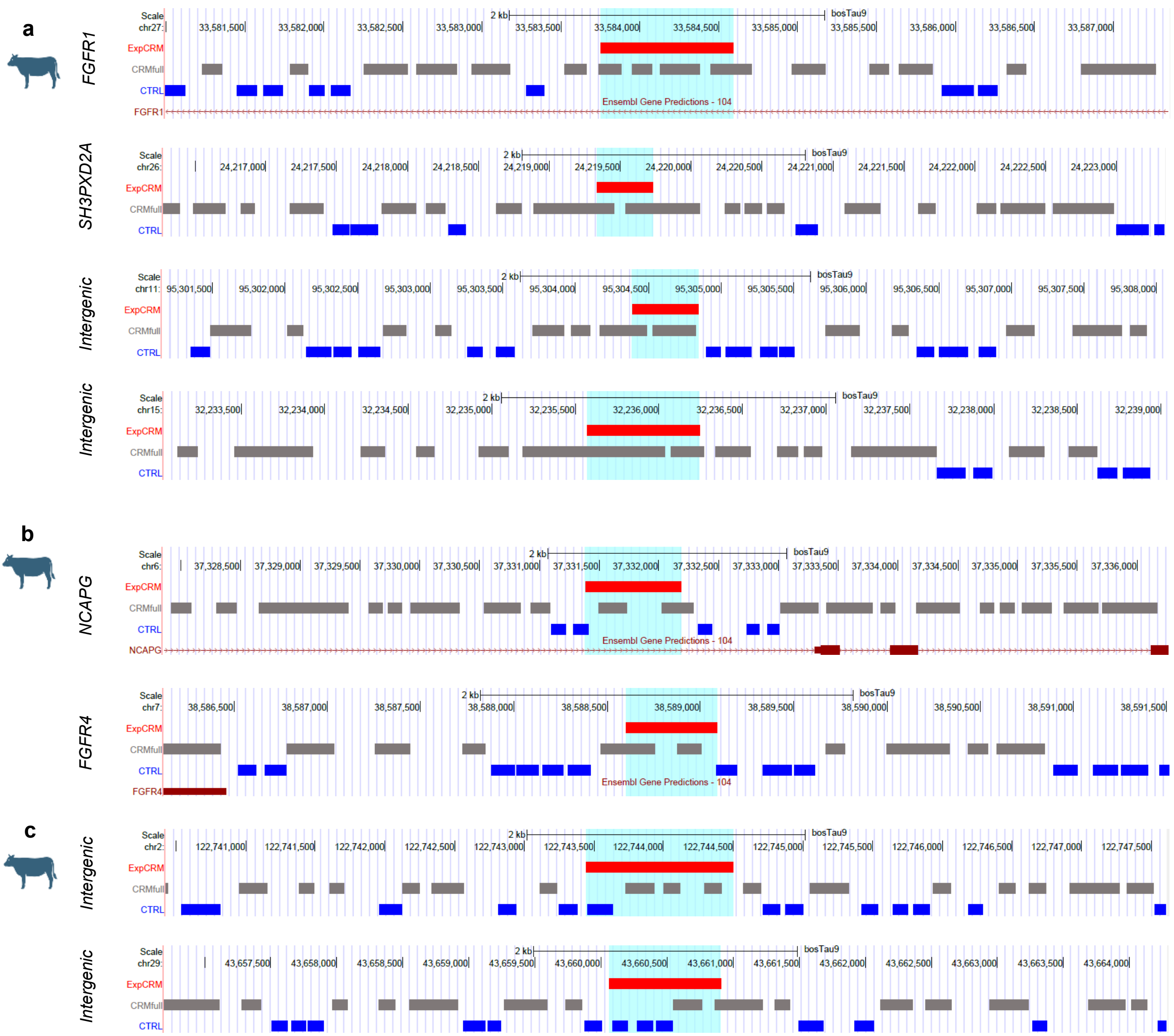

d Comparison of VRMOD CRM prediction performance with three state-of-the-art methods

Comparison	VRMOD	Ni et al. 2021 (P≤ 0.05)	EnhancerAtlas	ENCODE cCRE V3	ENCODE cCRE V4
Number of predicted CRMs	6,070,455	1,155,151	3,452,739	926,535	2,348,854
Genome coverage	42.00%	43.47%	58.78%	8.20%	20.39%
Mean length (bp)	213	981 (length increase as the parameter α increases)	680	273	268
Sensitivity (VISTA)	99.50%	100%	94.01%	85.99%	99.5%
