# Supplementary material for: Unlocking cis-regulatory landscapes across 500 million years of evolution and disease mechanisms
Source: NAR Genom Bioinform. 2026 Sep 15;8(3):lqag107. doi: 10.1093/nargab/lqag107 (PMC13575426; doi:10.1093/nargab/lqag107)
Supplement: Supplementary file 1 [file lqag107_supplemental_files.zip › Suppl_Figure4_VRMOD_VISTA_conservation_2024_12_06_Submitted.pdf]

Supplementary Figure 4

a hs52.2

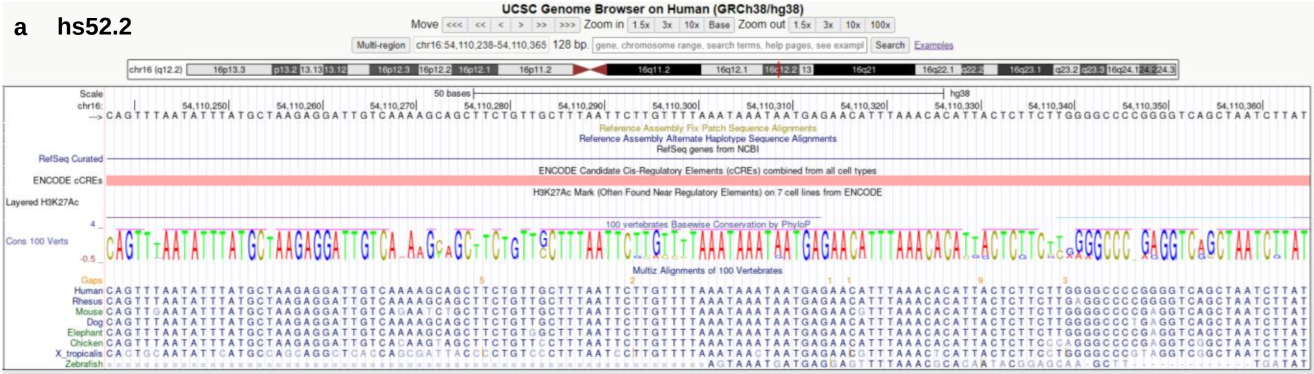

hs52.3

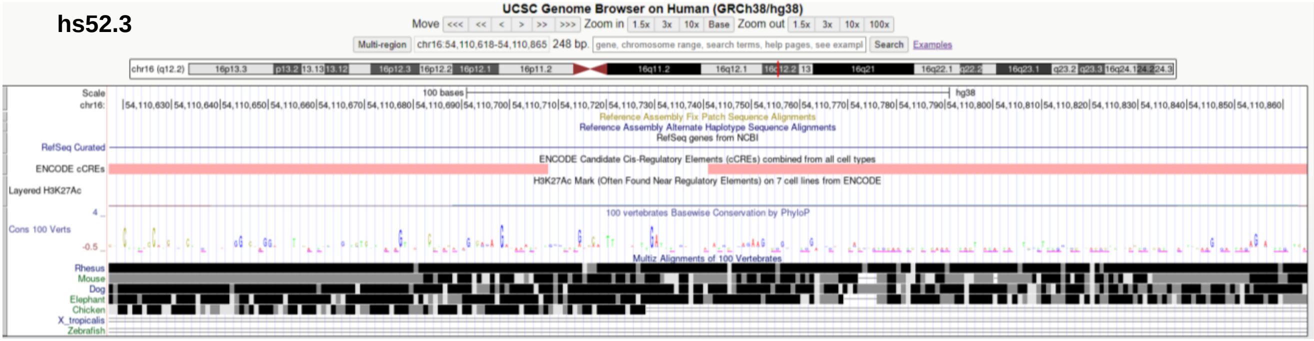

b

Species	Query coverage	E value	Percent identity
Macaca fascicularis (macaque)	100%	8e-98	93.55%
Bos taurus (cow)	98%	2e-28	74.12%
Capra hircus (goat)	99%	4e-26	73.83%
Ovis aries (sheep)	99%	2e-24	73.44%
Sus scrofa (pig)	42%	5e-10	75.47%
Spermophilus citellus (squirrel)	58%	3e-14	76.03%
Talpa europaea (mole)	54%	0.002	71.53%
