# Supplementary material for: Unlocking cis-regulatory landscapes across 500 million years of evolution and disease mechanisms
Source: NAR Genom Bioinform. 2026 Sep 15;8(3):lqag107. doi: 10.1093/nargab/lqag107 (PMC13575426; doi:10.1093/nargab/lqag107)

Supplementary Figure 6

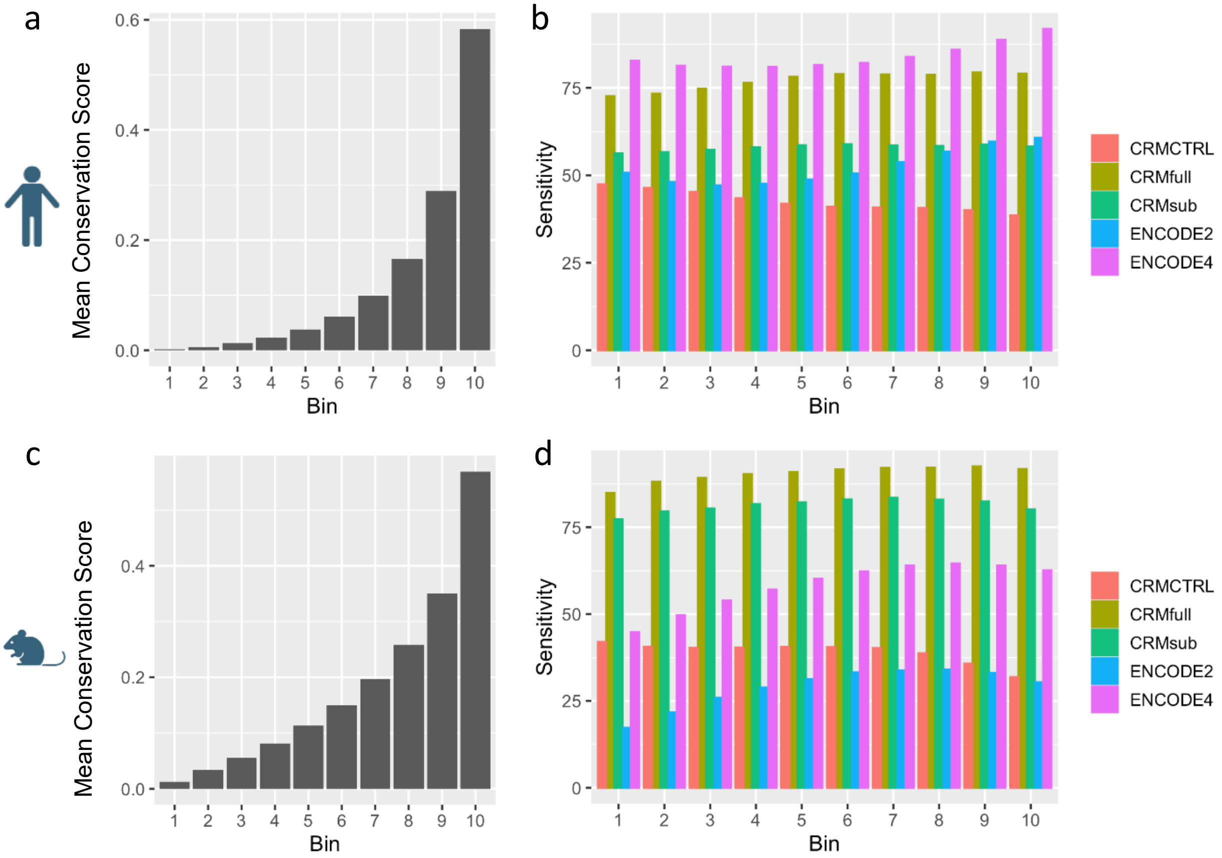

Supplement: Supplementary file 1 [file lqag107_supplemental_files.zip › Suppl_Figure6_VRMOD_CRM_Sensitivity_byConservationScore_2026_01_27.pdf]
