# Supplementary material for: Unlocking cis-regulatory landscapes across 500 million years of evolution and disease mechanisms
Source: NAR Genom Bioinform. 2026 Sep 15;8(3):lqag107. doi: 10.1093/nargab/lqag107 (PMC13575426; doi:10.1093/nargab/lqag107)
Supplement: Supplementary file 1 [file lqag107_supplemental_files.zip › Suppl_Figure8_VRMOD_Human_SNPs_evidence_2026_02_15.pdf]

Figure S8

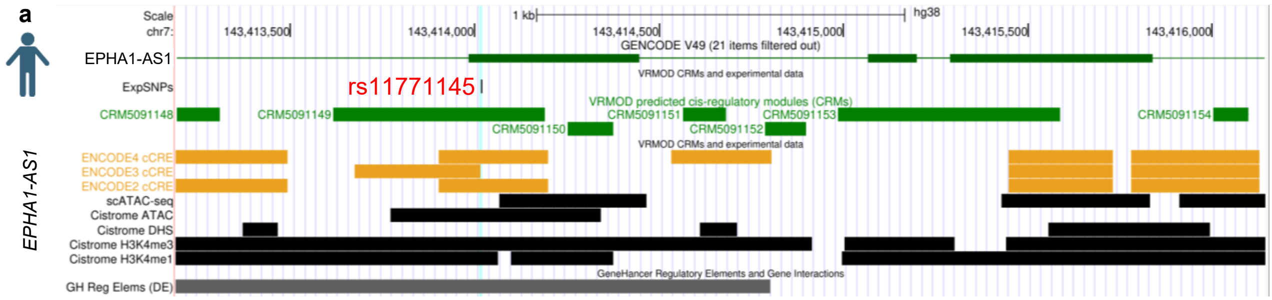
a
Scale
chr7:
1 kb
hg38
143,413,500
143,414,000
143,414,500
143,415,000
143,415,500
143,416,000
GENCODE V49 (21 items filtered out)
EPHA1-AS1
VRMOD CRMs and experimental data
ExpSNPs
rs11771145
VRMOD predicted cis-regulatory modules (CRMs)
CRM5091148
CRM5091149
CRM5091151
CRM5091153
CRM5091154
CRM5091150
CRM5091152
VRMOD CRMs and experimental data
ENCODE4 cCRE
ENCODE3 cCRE
ENCODE2 cCRE
scATAC-seq
Cistrome ATAC
Cistrome DHS
Cistrome H3K4me3
Cistrome H3K4me1
GeneHancer Regulatory Elements and Gene Interactions
GH Reg Elems (DE)
EPHA1-AS1

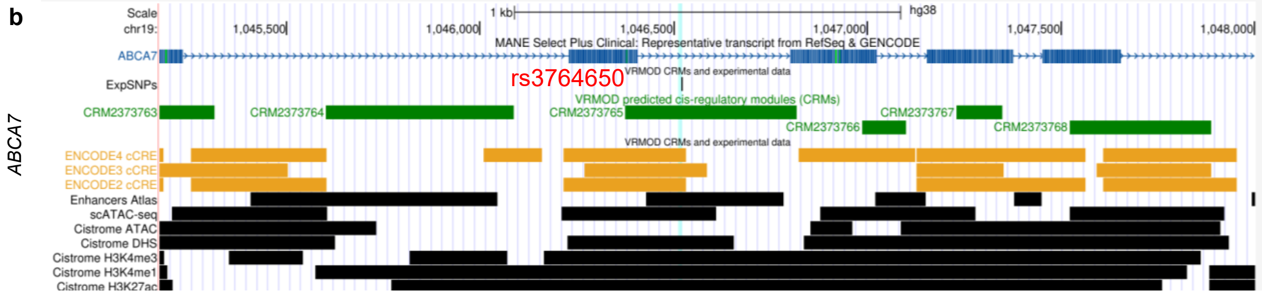
b
Scale
chr19:
1 kb
hg38
1,045,500
1,046,000
1,046,500
1,047,000
1,047,500
1,048,000
MANE Select Plus Clinical: Representative transcript from RefSeq & GENCODE
ABCA7
VRMOD CRMs and experimental data
ExpSNPs
rs3764650
VRMOD predicted cis-regulatory modules (CRMs)
CRM2373763
CRM2373764
CRM2373765
CRM2373767
CRM2373766
CRM2373768
VRMOD CRMs and experimental data
ENCODE4 cCRE
ENCODE3 cCRE
ENCODE2 cCRE
Enhancers Atlas
scATAC-seq
Cistrome ATAC
Cistrome DHS
Cistrome H3K4me3
Cistrome H3K4me1
Cistrome H3K27ac
ABCA7
