# Supplementary material for: Unlocking cis-regulatory landscapes across 500 million years of evolution and disease mechanisms
Source: NAR Genom Bioinform. 2026 Sep 15;8(3):lqag107. doi: 10.1093/nargab/lqag107 (PMC13575426; doi:10.1093/nargab/lqag107)
Supplement: Supplementary file 1 [file lqag107_supplemental_files.zip › VRMOD_Revision4_Supplementary_Fig_Ledgend_Final.docx]

**Supplementary Figure Legends**

**Supplementary Figure 1: Phylogenetic tree of the 309 vertebrate genomes available in the Ensembl database.**

**Supplementary Figure 2: Evaluation of VRMOD CRM prediction performance and alternative strategy of obtaining control regions for a fair evaluation. a**) Odds ratios and p-values comparing the sensitivity between CRMsub and CTRLs at 50%-overlap cutoff for mouse and human genomes. **b)** Schematic of alternative strategy of obtain control regions and predicted CRMs for a fair comparison. **c)** Comparison of the distributions relative to the genomic features between CRMsub and CTRLs obtained through alternative strategy for the mouse (left) and human (right) genome respectively. **d-e)** Odds ratios and p-values comparing the sensitivity between alternative CRMsub and CTRLs at 1bp-overlap cutoff (**d**) or 50%-overlap cutoff (**e**) for mouse and human genomes respectively. Gray bar: predicted CRMs. Blue bar: CTRL regions.

**Supplementary Figure 3: Comparison of bovine predicted CRMs with experimentally defined modules (ExpCRMs) with different relative positions to the adjacent CTRL regions. A)** Comparison between bovine CRMfull with four ExpCRMs that did not overlap with CTRL regions. **b)** Comparison between bovine CRMfull with two ExpCRMs that overlapped with adjacent CTRL regions at 1 bp-overlap cutoff. **c)** Comparison between bovine CRMfull with two ExpCRMs that overlapped with adjacent CTRL regions at 50%-overlap cutoff. **d**)

Comparison of VRMOD CRM prediction performance with three state-of-the-art methods. Red bar: experimentally defined CRMs; gray bar: predicted CRMs. Blue bar: CTRL regions; red box and line: gene models.

**Supplementary Figure 4: Conservation of the human predicted CRMs that were experimentally validated *in-ovo*. a)** Genome Browser image showing that CRM hs52.2 was highly conserved throughout the evolution with 93% nucleotide identity between human and chicken genome. The hs52.3 element showed no detectable homology between human and chicken genome. **b)** Table comparing the query identity, e-value, and percent identity of the hs52.3 element with macaque, cow, goat, sheep, pig, squirrel, and mole genomes. No significant sequence similarity was detected in the chicken or mouse genome.

**Supplementary Figure 5: VRMOD prediction of human non-conserved CRMs. a)** Two human non-conserved silencers located upstream of *YJU2B* or downstream of *LINC00658*. **b)** Two human non-conserved enhancers located upstream *NCF1* or *DBH*.

**Supplementary Figure 6: Evaluation of the impact of sequence conservation on CRM prediction sensitivity.** a, c) Mean conservation score of snATAC-seq peaks in each conservation score bin for human (a) and mouse (c) snATAC-seq peaks used for evaluation. b, d) Prediction sensitivity of human (b) and mouse (d) CTRL, CRMfull, CRMsub, ENCODE2, and ENCODE4 cCREs for snATAC-seq peaks in each bin at 50% overlap cutoff.

**Supplementary Figure 7: Evaluation of the impact of sequence GC content on CRM prediction sensitivity.** a, c) Mean GC content of human (a), mouse (c), and zebrafish (f) snATAC-seq peaks in each bin. b, d) Prediction sensitivity of human (b) and mouse (d) CTRL, CRMfull, CRMsub, ENCODE2, and ENCODE4 cCREs for snATAC-seq peaks in each GC content bin at 50% overlap cutoff. e) Comparison of the distributions between CRMsub and CTRLs relative to the genomic features for the zebrafish genome. f) Mean GC content of zebrafish snATAC-seq peaks in each bin. g) Prediction sensitivity of zebrafish CTRL, CRMfull, and CRMsub for snATAC-seq peaks in each GC content bin at 50% overlap cutoff. (h) Comparison of mean GC content of CRMsub and CTRL in each genomic features category for the zebrafish genome.

**Figure S8: Identification of experimentally validated single nucleotide polymorphisms (SNPs) associated with neurodegenerative diseases. a)** Genomic view of rs11771145, an experimentally validated SNPs (ExpSNPs) linked to AD in the *EPHA-AS1* locus. **b)** Genomic view of rs3764650, an ExpSNPs linked to AD in the *ABCA7* locus.

**Supplementary Video 1-4**: Whole embryo tissue clearing and 3D viewing of mCherry expression driven by the hs52, hs52.1, hs52.2 and hs52.3 element at embryonic stage 16 to 17 chick 24 hours after electroporation. **1)** hs52 -mCherry reporter expression were distributed across various locations in the developing embryo including liver and sympathetic chain. **2)** hs52.1-mCherry reporter expression were localized to the sympathetic chain, gut and pharyngeal arches. **3)** hs52.2-mCherry reporter expression were localized to the stellate ganglia and the developing heart. **4)** and hs52.3 -mCherry reporter expression were detected along the sympathetic chain, the gut and the liver.
